# What can we learn from COVID-19?: examining the resilience of primary care teams

**DOI:** 10.3389/fpsyg.2023.1265529

**Published:** 2023-11-22

**Authors:** Ashley M. Hughes, Kelley Arredondo, Houston F. Lester, Frederick L. Oswald, Trang N. D. Pham, Cheng Jiang, Sylvia J. Hysong

**Affiliations:** ^1^Department of Biostatistics and Epidemiology, University of Illinois at Chicago, Chicago, IL, United States; ^2^Center of VHA Innovation for Complex, Chronic Healthcare, Edward Hines JR VA Hospital, Hines, IL, United States; ^3^Department of Medicine, Baylor College of Medicine, Houston, TX, United States; ^4^Center for Innovations in Quality, Effectiveness, and Safety, Michael E. DeBakey VA Medical Center, Houston, TX, United States; ^5^Veterans’ Health Administration Office of Rural Health’s Veterans Center, White River Junction, VT, United States; ^6^VA South Central Mental Illness Research, Education and Clinical Center (SC MIRECC), a Virtual Center, Houston, TX, United States; ^7^Department of Management, University of Mississippi, Oxford, MS, United States; ^8^Department of Psychological Sciences, Rice University, Houston, TX, United States

**Keywords:** teams, team resilience, COVID-19, primary care, team performance, team member fluidity

## Abstract

**Introduction:**

The COVID-19 pandemic continues to place an unprecedented strain on the US healthcare system, and primary care is no exception. Primary care services have shifted toward a team-based approach for delivering care in the last decade. COVID-19 placed extraordinary stress on primary care teams at the forefront of the pandemic response efforts. The current work applies the science of effective teams to examine the impact of COVID-19—a crisis or adverse event—on primary care team resilience.

**Methods:**

Little empirical research has been done testing the theory of team resilience during an extremely adverse crisis event in an applied team setting. Therefore, we conducted an archival study by using large-scale national data from the Veterans Health Administration to understand the characteristics and performance of 7,023 Patient Aligned Care Teams (PACTs) during COVID-19.

**Results:**

Our study found that primary care teams maintained performance in the presence of adversity, indicating possible team resilience. Further, team coordination positively predicted team performance (B = 0.53) regardless of the level of adversity a team was experiencing.

**Discussion:**

These findings in turn attest to the need to preserve team coordination in the presence of adversity. Results carry implications for creating opportunities for teams to learn and adjust to an adverse event to maintain performance and optimize team-member well-being. Teamwork can act as a protective factor against high levels of workload, burnout, and turnover, and should be studied further for its role in promoting team resilience.

## Introduction

Since the declaration of a global pandemic in 2020, COVID-19 has wreaked havoc on public health, health policy, and the global economy (U.S. Department of Labor: Bureau of Labor and Statistics, 2021; World Health Organization, 2021), presenting workers and work teams with unprecedented challenges. The world response to contain the spread of COVID-19 has forever changed the way people work and live, making COVID-19 a type of adversity (i.e., a threat to entities, such as individuals, teams, and/or organizations’ performance or well-being; Kennedy et al., 2016; Hartmann et al., 2020). Faced with adversity of this magnitude, work teams must exhibit resilience to successfully sustain team performance (Alliger et al., 2015; Hartmann et al., 2020).

In line with Alliger et al. (2015), we define *team resilience* as the capacity of teams to manage challenges that threaten their performance and well-being. During times of adversity, such as COVID-19, teams must work together to “manage pressure effectively across the team as a whole” (Flint-Taylor and Cooper, 2017, p. 130). This captures two key characteristics of team resilience: (1) teams experience adversity as a collective challenges team performance or well-being, and (2) team properties preserve performance or help teams “bounce back” from adverse events (Hartmann et al., 2020). Theories that seek to describe and explain team resilience characterize the roles of inputs (e.g., organizational culture), mediators (e.g., processes), and output(s) that affect how teams overcome adversity and adjust in subsequent performance cycles (Alliger et al., 2015; Maynard et al., 2015; Bowers et al., 2017; Hartwig et al., 2020; Stoverink et al., 2020), such as those inherent to COVID-19 responses. However, team resilience theories have not yet been tested under the adversities introduced by the COVID-19 pandemic (e.g., constant changes in patient volume, working conditions, availability of personal protective equipment, policy guidance, and health risk to workers).

The central goal of this study is to determine the extent to which teams maintain performance during adverse conditions of COVID-19, intending to generalize a model and pattern of relationships for team resilience to similar adverse circumstances. We further examine how team performance may be helped or hindered by *team-member fluidity* (i.e., the dynamic flow of members entering and exiting teams; Bedwell, 2019), with a downstream impact on coordination and team performance. More specifically, our paper addresses the following research questions, using a large national sample of primary health care teams:

Research Question 1: Do known relationships between team resilience and team performance hold true in healthcare teams during the COVID-19 pandemic?

Research Question 2: Overall, does team-member fluidity facilitate or hinder team performance?

**Figure 1:**
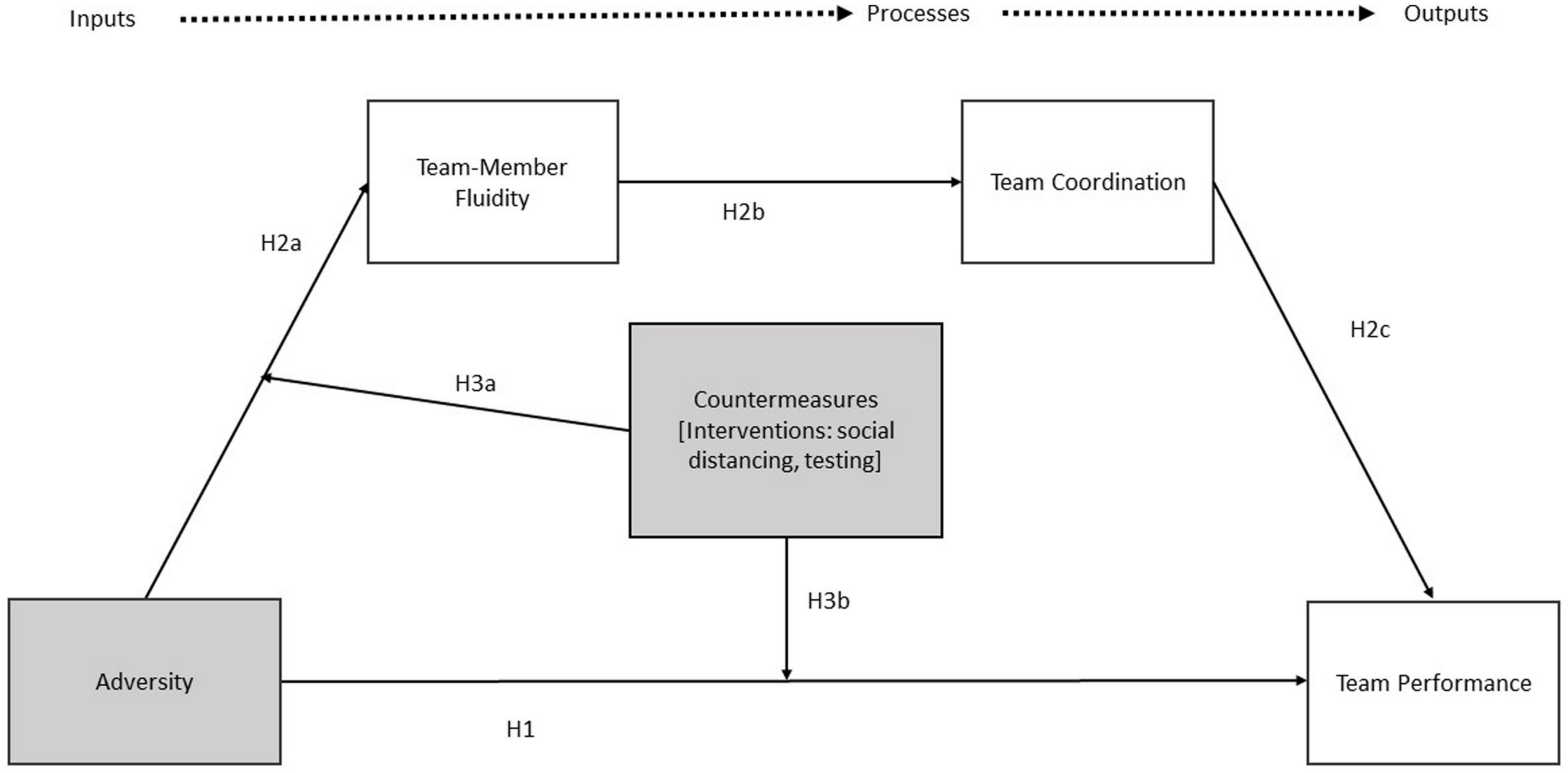
Healthcare team resilience framework considering COVID-19.

For the purpose of this paper, team resilience refers to an emergent state (Alliger et al., 2015; Stoverink et al., 2020; Brykman and King, 2021) which enables teams to ‘bounce back’ in the presence of an adverse stimulus. Teams which exhibit resilience will maintain or improve performance in response to an adversity (Bowers et al., 2017). In this context, our paper extends team resilience theory to create a testable model of team resilience during circumstances imposed by COVID-19 (see Figure 1). This in turn furthers the objective to empirically test how “teams in the wild” adjust their performance in the face of adversity. Drawing on this team resilience literature, we posit that under conditions of extreme adversity, such adversity decreases team performance in brittle (i.e., non-resilient) teams whereas resilient teams maintain performance levels. The current paper advances that effects to team performance occur both directly and indirectly, in that the direct effect of adversity influences team performance and adversity influences team performance through indirect effects explained by team-member fluidity and team coordination. We further posit these effects can be mitigated, however, through effective countermeasures. Using primary health-care teams as our population of interest, we describe the unique features of this team population that make it suitable for examining the extreme adversity of the COVID-19 pandemic in the following sections; we then review the team resilience literature to describe in more detail the predictions under conditions of extreme adversity.

### Adaptation or resilience: Is a stressor by any other name still stressful?

Successful teams must often maintain or even improve their performance when faced with challenges in the workplace. As researchers investigate how teams overcome or “bounce back” from these challenges, they can rely on the literature for two relevant mechanisms: team adaptation and team resilience (Kennedy et al., 2016). *Team adaptation* refers to a process involving teams’ adjustments to changing circumstances and the results of these adjustments (Christian et al., 2017). Teams adapt in the presence of adaptive stimuli which are largely viewed as changes or stressors internal to the team, such as changes in team composition (e.g., rotating team membership) or changes to the team’s task (Maynard et al., 2015; Christian et al., 2017). However, when teams encounter an unexpected and stressful event which originates *externally* to the team that necessitates team response (e.g., natural disaster, organization merger), team adaptation may fail to capture the full extent of how teams overcome the adverse event (Maynard et al., 2015).

The second mechanism, *team resilience*, reflects a team’s ability to positively adjust in the face of adversity to maintain or enhance performance. Given this definition, the conceptual overlap between team resilience and team adaptation is noteworthy (Maynard et al., 2015; Kennedy et al., 2016). Both sets of literature rely heavily upon the considerations for a “stressor” that prompts a team response (“adaptive stimuli” in team adaptation literatures or “adversity,” which prompts team resilience) and the nature of the adjustment in team process to be made. Following the Input-Mediator-Output–Input team process model (IMOI; Ilgen et al., 2005), as a team adapts during an adverse event, resiliency increases, which can in turn enhance a team’s ability to adapt, creating a cyclical process of potential improvement (Kennedy et al., 2016).

### Teams situated within the healthcare context

Organizational conditions shape work teams and are therefore an important consideration for understanding team resilience (Hartwig et al., 2020) and other team phenomena (Salas et al., 2015). In the healthcare sector, dynamic and complex organizational conditions create circumstances that either enhance or fragment team structures (Crawford et al., 2019; Hysong et al., 2019) and the execution of team attitudes, behaviors, and cognitions (ABCs) necessary for working together as an effective team. Because organizational conditions change over time, constant adaptation to those conditions is necessary for effective team performance (Gregory et al., 2021), especially in frontline healthcare teams, as has been appreciated prior to the pandemic (Fiscella and McDaniel, 2018; Gregory et al., 2021). With COVID-19’s global emergence, the healthcare workforce experienced an unpreceded increase in the volume and health risk of their workload (DeVoe et al., 2020), while concurrently adapting rapidly to changes in the mode of healthcare service delivery (e.g., rapid uptake of telehealth). Acting as a novel, disruptive, ubiquitous, and critical event, the COVID-19 pandemic changed how healthcare teams behaved, requiring them to adjust or create new behaviors to maintain performance (Morgeson et al., 2015). Taken together, the impact of COVID-19 on the work performed by healthcare personnel necessitates resilience possibly coupled with adaptive processes in teams to maintain function and provision of preventative healthcare services.

### The VHA primary care team setting

Primary care acts as the nexus of the US healthcare system, saving people’s lives on the front lines of COVID-19 via early diagnosis, reporting, and prevention. Primary care also provides continuity of care, accessibility to health services, public health message delivery, counseling, and treatment for chronic health problems (National Academies of Sciences, Engineering, and Medicine, 2021). Yet, the state of the science of team effectiveness in primary care remains largely underexplored, despite multiple calls for empirical evidence in the area (Marlow et al., 2017; Fiscella and McDaniel, 2018).

In the last decade, primary care services have shifted toward a formalized team-based model for delivering outpatient care (Fiscella and McDaniel, 2018). In the Veterans Health Administration (VHA), the team-based model of primary care delivery is structured around an interprofessional core team comprising a primary care provider (PCP – usually a physician, though occasionally a physician’s assistant or advanced practice nurse), registered nurse, medical assistant, and administrative clerk. This clinical primary care team configuration is known within the VHA as *Patient Aligned Care Teams (PACTs)* that are assigned a patient panel of approximately 1,200 patients (American Academy of Family Physicians, 2008) – each patient is assigned to a core PACT in lieu of an individual PCP. Since the declaration of the pandemic in 2020, the successful resilience of primary care has become essential to maintaining the cornerstone of preventative services in the US healthcare system.

### Healthcare at the forefront of the pandemic response

The COVID-19 pandemic serves as an important and novel context for understanding teams in extreme conditions (Morgeson et al., 2015). During the COVID era, individuals, teams, and organizations face unprecedented challenges on how to adjust to a “new normal” (Tannenbaum et al., 2021; Heavner et al., 2023). Crises are among the types of potential stressors that influence or incite team resilience, and thus, PACTs serve as an ideal natural laboratory for testing our proposed model (Alliger et al., 2015). As an unexpected and novel trigger, COVID-19 has forced healthcare teams to adopt new ways of operation, such as mobilizing scant resources (e.g., personnel, Personal Protective Equipment [PPE], and COVID tests) and keeping up to date with information on a rapidly evolving scenario (DeVoe et al., 2020; Krist et al., 2020; Heavner et al., 2023). Considering the importance of resilience during the pandemic, we leverage models of team resilience, team-member fluidity, team adaptation, and team performance (Weaver et al., 2013; Hartmann et al., 2020; Hartwig et al., 2020; Gregory et al., 2021; Tannenbaum et al., 2022) to advance a theoretical model for team resilience in ‘crisis’ events for team-based primary care (see Figure 1).

### Unpacking resilience: How adversity affects team performance

Employees and teams have been performing in a prolonged state of uncertainty since the initial declaration of the COVID-19 pandemic as a national emergency on March 13, 2020 (The White House, 2021). During this public health crisis, healthcare workers experienced depletion of necessary physical and psychological job resources (e.g., PPE, staffing, self-efficacy, co-worker or supervisor support) and increased job demands (e.g., workload, working duration, and financial pressure; Cohen et al., 2020); this imbalance contributes significantly to burnout in healthcare professionals (Chandrasekar and Ng, 2017; Helfrich et al., 2017; Heavner et al., 2023).

*Adversity* serves as the stressor external to the team that elicits a resilient response. Types of stressors theorized to trigger resilience encompass various challenges teams face, including but not limited to *time pressure*, *hazardous work,* and *‘crisis’ events* (Alliger et al., 2015, p. 177). Although the concept has not been unambiguously defined, *team resilience* generally refers to processes of “managing pressure effectively across the team as a whole [. . .], that further strengthen the capacity of the team to deal with future challenges in adversity” (Flint-Taylor and Cooper, 2017, p. 130). Environmental adverse events can have an impact at the individual, team, and organizational level (Morgeson et al., 2015). Therefore, an adverse event such as COVID-19 presents stressors at multiple levels that threaten team performance. Beyond the COVID-19 context, adversity can induce loss, which in turn initially diminishes team performance. Stressors from COVID-19 accumulate, generating enormous demands while the resources necessary to sustain and improve job performance are insufficient to compensate (DeVoe et al., 2020; Heavner et al., 2023). However, medical teams may be well-suited to rapidly adjust to multiple stressors, given the ongoing need for adjustment and adaptation over time, which may in turn strengthen or build the capacity for resiliency in the face of adverse crisis events, such as the introduction of COVID-19. As such, maintaining levels of performance becomes more difficult.

*Hypothesis* 1: Healthcare teams exhibit resiliency such that team performance is maintained in the presence of intense adversity.

#### Team fluidity and coordination explain effects of adversity on team performance

The emergence of team resilience is complex, often explained through processes known to explain relationships between stressors and performance (Brykman and King, 2021). In the case of healthcare teams during the COVID-19 pandemic, adversity may trigger a cascade of disruptions unanticipated by the team, triggering internal team adaptations which explain changes in team performance. Team continuity (i.e., low team-member fluidity or less turnover within a team) within the core primary care team structure is often necessary to meet stated healthcare team objectives (e.g., continuity and coordination of care; American Academy of Family Physicians, 2008). Although team-member fluidity (i.e., the rotation of team members in and out of a care team) is multi-dimensional (Arrow and McGrath, 1993) and common in particular healthcare team types (Andreatta, 2010; Hughes et al., 2016), team-member fluidity can be more prevalent during a crisis (Tannenbaum et al., 2021; Traylor et al., 2021). In fact, healthcare teams that experience turnover are associated with a 67% increase in the odds of a remaining team member experiencing burnout (Helfrich et al., 2017). Simultaneously, COVID-19 introduces an influx of stressors (e.g., contracting COVID) and decreases available job resources; as such, primary care teams face tremendous uncertainty (e.g., clinic closure) and change, exacerbating the potential for team-member burnout and subsequent turnover. Due to such experiences, healthcare teams may experience significant team-member fluidity through turnover during adverse crisis events, such as the COVID-19 pandemic.

Team member fluidity, particularly loss or turnover of a team member, can create negative downstream implications for teamwork by disrupting the shared cognitions of a team (e.g., transactive memory systems, shared mental models; Tannenbaum et al., 2012; Bedwell, 2019). Namely, team coordination – which serves as the lynchpin of behavioral team processes- may be particularly vulnerable during periods of team member fluidity (Peltokorpi, 2008) as its execution relies upon accurate and in-tact team knowledge structures (DeChurch and Mesmer-Magnus, 2010). The behavioral process of team coordination involves sequencing and timing team member’s interdependent actions which are then assembled at the team level (Marks et al., 2001). Healthcare team coordination manifests in the completion of interdependent clinical tasks (Schmutz et al., 2019), including successful patient resuscitation (Marsch et al., 2004; Yamada et al., 2016) and timely administration of medications (Siassakos et al., 2011). Changes in healthcare team membership incite changes in team knowledge structures which may subsequently impact coordination. Taken together, we anticipate these mechanisms of team-member fluidity to negatively impact coordination and collectively explain decrements in team performance.

*Hypothesis* 2: Team resilience (i.e., the relationship between adversity and team performance) is explained by both team-member fluidity and team coordination, such that teams experiencing: (a) greater adversity experience increases team-member fluidity (i.e., team turnover), (b) greater team-member fluidity see diminishes team coordination and (c) diminished team coordination experience reduced team performance.

#### Moderating role of community countermeasures

COVID-19 is a highly contagious and deadly virus, and for over a year before the first vaccines were distributed in December 2020 (Florko, 2020), effective public health interventions (referred to as community-level countermeasures) offered the sole defense against viral spread. Non-pharmaceutical countermeasures which included testing, masking, and social distancing policies (Kenyon, 2020) were often implemented ineffectively by US state governments (Hale et al., 2020). As a result, implementation of these countermeasures was variable across states, having a weak national effect on controlling the rate of COVID-19 infections over time (The White House, 2021). Public health interventions may serve to slow the spread of the virus, thereby lessening the intensity of adversity experienced by frontline care providers (Tannenbaum et al., 2022). Primary care teams serving communities with enforced public health countermeasures may have greater capacity to mend, recover, and adjust strategies which promote greater resilience in the face of stressors (Tannenbaum et al., 2022). In this way, public health interventions may act as a community-level countermeasure preserving primary care team function, promoting resilience.

*Hypothesis* 3a: Community-level countermeasures (e.g., social distancing, mask mandates, and testing) moderate the relationship between adversity and team-member fluidity (i.e., turnover), such that the extent of exposure to community countermeasures attenuates the impact of adversity on team-member fluidity.

*Hypothesis* 3b: Community-level countermeasures (e.g., social distancing, mask mandates, and testing) moderate the relationship between adversity and team performance, such that the extent of exposure to community countermeasures attenuates the level of team resilience exhibited (i.e., effects of adversity on team performance).

## Method

### Participants and setting

As part of a larger study (Hysong et al., 2019), we conducted an archival study sampling 27,753 primary healthcare personnel representing 7,023 PACTs with a median team size of 3 members (*M* = 3.9, *SD* = 0.67, 2–10) that deliver care at 152 VHA healthcare facilities nationwide, including VA Medical Centers (VAMC) and Community-Based Outpatient Clinics (CBOCs).

The VHA is the largest integrated healthcare system in the United States, providing care to nearly 10 million veterans annually, and employing over 322,030 full-time health care professionals and support staff at 1,255 health care facilities. The VHA is an ideal setting to conduct this research for several reasons. First, VHA provides monthly reports on the members within each healthcare team, thus allowing for the calculation of monthly changes in team turnover (Crawford et al., 2019). Additionally, all VAMCs nationwide have used the same electronic health record (EHR) for over two decades; clinical data are uploaded into a national Corporate Data Warehouse (CDW). team and individual employee data are also available from centralized sources, with identifying fields available to link all sources into a single dataset for analysis. Thus, the VHA setting affords a unique and important opportunity to study team-member fluidity with sufficient sample size for multi-level quantitative analysis. For this study, we obtained data from April to June in 2020. This timeframe reflects the extreme forms of adversity (i.e., start of the global pandemic) and the nature of teams’ response to this adversity.

### Measures

Unless otherwise noted, all measures described below were obtained from multiple databases curated and maintained by the VHA in their CDW, a national repository of data from the Veterans Health Information Systems and Technology Architecture (VistA) and other VHA clinical and administrative systems. For ease of reading, we present the operationalization of measures and list the data sources from which the measures were drawn in Table 1.

**Table 1:** Variables and operational definitions within the model.

Level of analysis	Variable in model	Definition	Data source	Measure extracted	Month
Organization	Adversity^a^	Number of COVID-19 cases reported monthly in each VAMC divided by the number of unique veteran patients served annually by the facility	Department of Veterans Affairs COVID-19 National Summary	COVID-19 Case Rates	April
	Community Countermeasures^b^	Weighted composite of 12 community variables; ^1^ PVI possible values range from 0 to 1, a higher PVI score reflects higher risk due to lower countermeasure implementation	NIEHS Pandemic Vulnerability Dashboard	Pandemic Vulnerability Index (PVI)	April
Team	Team-Member Fluidity^c^	Dichotomized to reflect whether a team member left a team within a given month	VA Corporate Data Warehouse (CDW), PACT Compass, and Team Assignments Report (TAR)	Team Turnover *	May
	Team Performance^c^	The total number of ER/Urgent Care encounters for assigned primary care patients in the last 12 months divided by the team assignments.		ER/Urgent Care Utilization	June
	Team Coordination^d^	Percent of patients in a team’s patient panel (i.e., the number of patients under the care of a given team) observed during the period of interest (in this case, month-long cohorts) with BP readings of 140/90 or less at the time of their visit	eQM Measures	Hypertension Management^2^	May

Grey rows denote level-2 variables aggregated to station level and white rows denote level-1 variables aggregated to the team level.^a^Data extracted from Department of Veterans Affairs COVID-19 National Summary.^b^Data extracted from NIEHS Pandemic Vulnerability Dashboard.^c^Data extracted from VA Corporate Data Warehouse (CDW), PACT Compass, and Team Assignments Report (TAR).^d^Data extracted from Electronic Quality Measures (eQM) Measures.*Calculated using dichotomous variable determining whether a team experienced turnover within a given month. ^1^Marvel et al. (2021); ^2^ Hysong et al. (2016).

#### Adversity

Team exposure to adversity represents a defining element of the team resilience process (Stoverink et al., 2020). The chief stimulus of interest in our study is the adversity teams experience through their collective experience during COVID-19, which has varied markedly by geographic region over the course of the pandemic. As adversity may vary in intensity (Hartmann et al., 2020), thus impacting the team’s collective response, we defined adversity as the number of COVID-19 cases reported monthly in each VAMC divided by the number of unique veteran patients served annually by the facility. We obtained this data from the Department of Veterans Affairs COVID-19 National Summary (U.S. Department of Veterans Affairs, 2020) and the VA team assignments table. Curated by VA’s Office of Analytics and Performance Integration, the COVID-19 National Summary tracks COVID-19 cases, hospitalizations, and deaths at every VA medical facility in the United States. Data are reported daily at the organizational level and made available to the public via VA’s national COVID-19 dashboard (U.S. Department of Veterans Affairs, 2020).

#### Community-level countermeasures

Countermeasures implemented at the community level were operationalized using the National Institute of Environmental Health Sciences’ (NIEHS) Pandemic Vulnerability Index (PVI; U.S. National Institute of Environmental Health Sciences, 2021). The PVI provides a weighted risk profile composite created from 12 variables covering four domains: infection rate (i.e., transmissible cases, disease spread), population concentration (i.e., population mobility, residential density), intervention (i.e., testing, social distancing), and health and environment (i.e., population demographics, air pollution, age distribution, co-morbidities, health disparities, and hospital beds; Motsinger-Reif, 2020); possible values range from 0 to 1. PVI as a composite score signifies the vulnerability of a geographical region to the spread of COVID-19 and is reported at the community (county) level, making the reverse score of PVI signify the extent to which community-level countermeasures were implemented to combat the spread of COVID-19. Data are reported at the county level and updated daily.

#### Team-member fluidity

Team-member fluidity describes the rotation of a team member—either entering or leaving a team—such that a change in team membership has occurred (Bedwell, 2019). The Team Assignments Report (TAR) is an administrative report housed within the CDW; TAR displays the names and roles for all active VA primary care workers, including to which PACT(s) they belong to at a given time, each month. Therefore, TAR is updated when an individual leaves a team due to reassignment, job turnover (i.e., left the health system due to termination or quitting), or death and is not an indicator of when team-members are temporarily out-of-office (e.g., vacation or sick leave). Team-member fluidity was extracted from this source by identifying whether a team-member change had occurred between 2 months. Ideally, PACTs would have a fully staffed team with no change in team membership (Andreatta, 2010). We therefore operationalize team-member fluidity by whether anyone left in given month. For our purposes, a separation from the team may mean leaving the facility or the VHA, or it could simply involve a member who transfers to a different team.

#### Team coordination

Behavioral markers offer an observable set of actions that represent team process (Flin and Martin, 2001). Behavioral marker(s) of coordination capture “sequence[d] interdependent taskwork” (Salas et al., 2008, p. 58); in healthcare, team coordination is often measured in the field by observing the output of the team’s coordinative acts (Schmutz et al., 2019; Dinh et al., 2020). Accurate blood pressure measurement and management places considerable coordination demand involving all core members of the primary care team (Benzer et al., 2016; Hysong et al., 2016; Fiscella and McDaniel, 2018). We therefore operationalized team coordination in terms of the teams’ ability to maintain their patients’ BP under control, specifically, the percentage of patients in a team’s patient panel (i.e., the number of patients under the care of a given team) observed during the period of interest (in this case, month-long cohorts) with BP readings of 140/90 or less at the time of their visit. Team coordination was extracted from VHA’s Electronic Quality Measures (eQM), which relies on nationwide, automated extraction of data pooled in the CDW, generating near real-time, full-population measures of clinical performance updated daily (O’Mahen et al., 2021). eQM scores are calculated using the entire patient population (100% sampling), thereby eliminating both sampling and missing-data concerns.

#### Team performance

Outcomes for healthcare teams are operationalized through patients’ health outcomes within the teams’ care (Hughes et al., 2016; Hysong et al., 2021; National Academies of Sciences, Engineering, and Medicine, 2021). Primary care, for instance, is charged with management of chronic and acute disease. Health concerns that could be addressed in primary care, if left unchecked, often result in trips to urgent care or the emergency department (ED; Coster et al., 2017), making high ED utilization an indicator of poor primary care team performance (Dowd et al., 2014; Crawford et al., 2019; Hysong et al., 2022). A body of literature has shown that primary care continuity and accessibility helped reduce rates of ED use (e.g., Pourat et al., 2015; van den Berg et al., 2016). As such, we operationalized team performance using ED/urgent care visits, where higher numbers of ED visits indicate worse primary care team performance. These data were extracted using “ER/urgent care utilization rate” from VHA’s PACT Compass, which gives primary care managers and staff access to data on key metrics such as access, continuity of care, and care coordination. The PACT Compass is updated nightly and created from fields within CDW.

### Procedure

#### Dataset integration

Data sources were combined using common data fields (e.g., team-member social security numbers, primary care team ID, 3-digit facility code, city, and state) to form a single database for analyses. Data were aggregated to the lowest common level of analysis. As data sources were collected and reported either monthly or daily, all data were aggregated to monthly reports. Similarly, data were aggregated to either station (i.e., a hospital and its satellite clinics) or team level.

#### Data diagnostics

Data were reviewed for completeness, quality, and assessed for outliers. First, data were screened to ensure that values fell within the plausible ranges (e.g., team sizes with an *n* > 1). Next, we identified an outlying facility with an undue influence on model results, based on having a Cook’s distance greater than two at either the team or facility level of analysis. Upon closer examination, the outlier represents a facility located in the Bronx, New York. Given the purpose of this study to examine team resilience during an adverse crisis event, and that New York was one of the hardest hit areas early in the pandemic (2020), this outlying case was determined to help the study meet objectives of understanding team resilience in high levels of adversity and was thereby retained.

#### Transparency and openness

We describe our sampling plan and all measures used in the study; all data are available with approved data use agreements through the original data sources reported herein. Analysis code and research materials are available upon request. The current study’s design and analysis were not pre-registered, as the overall project protocol was developed pre-COVID (Hysong et al., 2019). No data were excluded from the analysis and no manipulations occurred.

#### Analytic strategy

Two levels of analysis were used in this study with teams as the lowest level of analysis, consistent with the focus of the current research. Structurally and analytically, teams are nested within facility or “station” level (i.e., a VA Medical Center and its subsidiary satellite clinics). Except for team-member fluidity (i.e., dichotomous), all outcome variables were modeled under the assumption of having normally distributed residuals. Team-member fluidity was dichotomized to operationalize whether any team-member left a team within a given month, vs. having all team members being present. This dichotomization is justified, because having any turnover vs. no turnover at all is a qualitatively different phenomenon (and stressor) in teams, and because it is consistent with the data distribution (most teams remain intact each month). Moreover, this approach to scoring team-level turnover remains amenable to Bayesian estimation techniques and due to the large sample size, remains sufficiently powered to find multilevel turnover effects where they exist.

A multi-level path analysis was used to analyze the delayed effects of adversity on primary care team stability and performance, accounting for station-level effects and their influences on team-level characteristics (months used for each variable are reported in Table 1). Bayesian estimation was selected to estimate model fit and parameters, because a traditional analytic model using maximum likelihood estimation was not estimable (Depaoli et al., 2021). In particular, the use of a dichotomous mediating variable within a sequential mediation model, coupled with retention of the extreme outlier positions Bayesian estimation to provide more accurate and robust analyses. Data were analyzed using Mplus version 8.8 (Muthen and Muthen, 2012). We report the 95% credibility interval(s) (CrIs) and posterior predictive *p*-values (PPP) for all estimated models. Models with a PPP between 0.05 and 0.95 were considered to fit the data well (Gelman et al., 2003).

## Results

**Figure 2:**
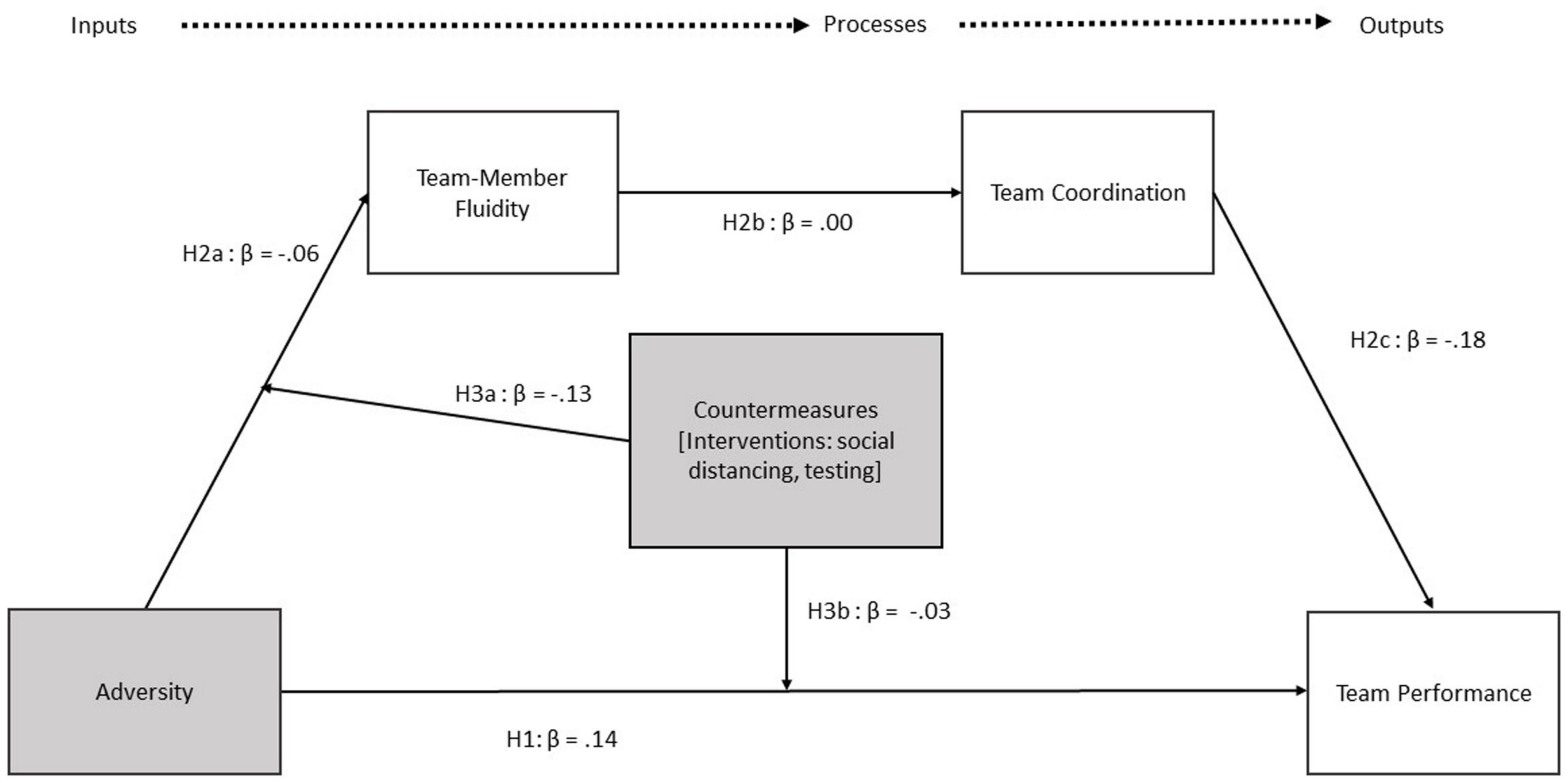
Healthcare team resilience framework with parameter estimates.

Participant and team characteristics of PACTs are reported in Table 2; variable means, standard deviations, and correlations are presented in Table 3. Model fit was evaluated using the PPP which fell within the acceptable range for all estimated models (see Table 4). Figure 2 summarizes the results of our analyses.

**Table 2:** Characteristics of VHA primary care team members and PACTs.

Characteristic	N	%
Individual-level Characteristics		
Race		
White	15,084	54.35
Black or African American	5,793	20.87
American Indian and Alaska Native	303	1.09
Asian	2,960	10.67
Multi-racial or Other/Multiple Race	903	3.25
Hispanic or Latino	1,164	4.19
Native Hawaiian/Other Pacific Islander	128	0.46
Undisclosed or missing	1,418	5.12
Sex		
Male	4,914	17.71
Female	17,440	62.84
Undisclosed	5,399	19.45
Role		
Primary Care Provider	6,882	24.79
Registered Nurse (RN)	7,636	27.51
Licensed Vocational Nurse (LVN)	6,670	24.04
Clerk	6,565	23.66
Team-level Characteristics		
Clinical Service Offerings	N	%
Primary Care only	3,783	53.87
Women’s Health	2,472	35.20
Other	768	10.93
Team Size N = 7,023	M = 3.9	SD = 0.67

Individual and team-level PACT characteristics reported for April 2020. Clinical service offerings (sometimes referred to as “Clinical Focus”) refer to services available through a PACT; “Other” category in Clinical Service Offerings includes Infectious Diseases, Spinal Cord Injury, Academic, Geriatric, Homeless, Post-Deployment Care, Renal/Dialysis, and Serious Mental Illness.

**Table 3:** Facility level means, standard deviations, and correlations between study variables with outlier.

Variable	M	SD	N	1	2	3	4	5
1. Adversity	0.03	1.02	106	-				
2. Mean Team Member Fluidity	0.18	0.14	130	0.00	-			
3. Mean Team Coordination	0.72	0.05	127	−0.08	0.04	-		
4. Team Performance	0.40	0.21	124	0.12	−0.01	0.04	-	
5. Countermeasures	−0.002	0.04	106	0.01	0.02	−0.21*	−0.07	-
Team level standard deviations and correlations between study variables with outlier								
Variable	M	SD	N	1	2	3		
1. Team Member Fluidity	-	0.35	7,736	-				
2. Team Coordination	-	0.10	6,228	0.00	-			
3. Team Performance	-	0.29	2,153	0.06*	−0.16***	-		

*p < 0.05; **p < 0.01; *** *p* < 0.001.

**Table 4:** Parameter estimates.

		With outlier				Without outlier			
				95% Credibility Intervals (CrIs)				95% Credibility Intervals (CrIs)	
Predictor	Outcome	Estimate (B)	S.D.	Lower	Upper	Estimate (B)	S.D.	Lower	Upper
Adversity	Team Performance	0.02	0.02	−0.01	0.06	0.02	0.02	−0.03	0.06
	Team-Member Fluidity	−0.03	0.06	−0.15	0.08	−0.20	0.07	−0.34	−0.05
Team-Member Fluidity	Team Coordination	0.000	0.002	−0.01	0.004	0.000	0.002	−0.01	0.004
Team Coordination	Team Performance	−0.53**	0.09	−0.69	−0.35	−0.52**	0.09	−0.69	−0.35
Mediator(s)	Relationship(s)	Estimate (B)	S.D.	Lower	Upper	Estimate (B)	S.D.	Lower	Upper
Team features (Team-Member Fluidity and Coordination)	Adversity to Team Performance	0.000	0.00	−0.001	0.001	0.000	0.001	−0.004	0.002
Moderator(s)	Relationship(s)	Estimate (B)	S.D.	Lower	Upper	Estimate (B)	S.D.	Lower	Upper
Countermeasures	Adversity to Team Performance	−0.09	0.38	−0.84	0.66	−0.06	0.56	−1.17	1.06
	Adversity to Team-Member Fluidity	−1.47*	1.11	−3.63	0.75	1.73*	1.43	−1.07	4.52

*p < 0.10; ** *p* < 0.05.

Hypothesis 1 predicted that teams would maintain performance in the presence of adversity. As seen in Table 4, results support this hypothesis in that primary care team performance did not change to a significant extent as a function of adversity, B = 0.02, *p* > 0.05, 95% CrI [−0.01, 0.06], β = 0.14.

Hypothesis 2 predicted team-member fluidity and team coordination would mediate the relationship between adversity and team performance. More specifically, Hypothesis 2 advanced three propositions to describe the nature of the expected relationships. Hypothesis 2a stipulated that adversity predicts increased team-member fluidity. Hypothesis 2a (B = −0.03, β = −0.06, *p* > 0.05, 95% CrI [−0.15, 0.08],) was not supported, showing a nonsignificant effect of adversity on team member fluidity opposite of the direction specified (i.e., team-member fluidity was less likely in areas with higher rates of COVID-19). Hypothesis 2b, which predicted that the presence of team-member fluidity decreased team coordination, was also not supported, as the estimated effect size for Hypothesis 2b was both weak and non-significant (B < 0.0005, β = −0.004, *p* > 0.05, 95% CrI [−0.005, 0.004]). Hypothesis 2c predicted that higher levels of team coordination positively predict team performance, which was supported (B = −0.53, β = −0.18, *p* < 0.01; 95% CrI [−0.69, −0.35]), meaning results provide partial support for Hypothesis 2.^1^ However, the overall indirect effect(s) evaluating the hypothesis (i.e., that team-member fluidity would play a role in mediating the relationship between adversity and team performance through team coordination) was not supported (B_indirect effect_ < 0.0005, 95% CrI [−0.001, 0.001], β < 0.0005).

Hypothesis 3 predicted that countermeasures would moderate the relationship(s) between (a) adversity and team-member fluidity, as well as (b) adversity’s relationship with team performance. Results partially support Hypothesis 3a (B = −1.47, *p* < 0.10, 95% CrI [−3.63, 0.75], β = −0.13) and fail to support Hypothesis 3b (B = −0.09, *p* > 0.05, 95% CrI [−0.84, 0.66], β = −0.03).

## Discussion

### Overview

We examined the extent to which teams exhibit resilience in the presence of intense adversity, such as the COVID-19 pandemic. Using one of the largest known samples of healthcare teams in the United States, this study tested the extent to which team-member fluidity impacts coordination and team performance as a result of the adversity a team experiences, making this the first known study to empirically test a model of team resilience. Contrary to expectations, primary care team performance did not significantly change as a function of the adversity. Among several factors proposed to influence team performance, team coordination was robust to team-member fluidity, yet positively predicted team performance. This in turn heightens the importance of bolstering team coordination efforts in the primary care arena, particularly during adverse crisis events. Community-level countermeasures may have been effective at reducing the strength of the adverse stimuli but did not play a statistically significant role in primary care team-member rotation in the current study. Team-member fluidity, as operationalized in our study, did not meaningfully predict changes to coordination or team performance. This finding contradicts current perspectives on team-member fluidity (Bedwell, 2019) and prompts further inquiry focused on *why* team-members are fluid.

At the time of this writing the pandemic lives on, arising in the form of new variants and continuing to spike in vulnerable populations across the world. Outcomes for team resilience in applied settings – particularly within healthcare- impact safety, quality, and efficiency, especially when stakes are high. Our study answers the longstanding call to action to better understand team resilience for ‘teams in the wild’ by examining resilience in a novel circumstance (Wildman et al., 2012; Salas et al., 2018; Traylor et al., 2021). This study used a national dataset consisting of 7,023 teams working in VA primary care healthcare facilities during the COVID-19 pandemic to advance knowledge of team adaptation in extreme scenarios, bearing practical and theoretical implications. We organize the theoretical implications by the main objectives of this study.

#### Do known relationships between team resilience and team performance hold true in healthcare teams during the COVID-19 pandemic?

Team resilience theories suggest an initial “dip” in team performance may present prior to team adjustment and the emergence of resilience. However, theories are unclear as to *when* performance maintenance and/or improvements should occur in response to adverse events. Furthermore, past research on resilience suggests a lack of conceptual clarity in team resilience. It does this in that team resilience may be operationalized as a process (Bowers et al., 2017) or as an outcome and the distinctions between team adaptation and resilience are muddled at best as team adaptation may enable team resilience (Kennedy et al., 2016). Study results did not show a significant relationship between team performance and adverse events. In fact, healthcare teams maintained both their coordination and their performance. As healthcare team adaptation serves as the hallmark of an effective medical team (Gregory et al., 2021), this could suggest that healthcare teams were resilient at the beginning of the COVID-19 pandemic via coordination, which significantly predicted performance. It is also possible that the intensity of the adversity alone does not impact team performance. Rather, factors (e.g., full staffing) not accounted for in our theoretical model may shield primary care teams from extreme process loss (Bedwell et al., 2012). Ways to regularly capture and record other types of teamwork should be explored, such that the mechanisms through which they are protected during times of extreme crisis can be better understood.

#### Does team-member fluidity facilitate or hinder team performance?

Team-member fluidity, as operationalized by team turnover, did not significantly predict coordination nor team performance. Views on team-member fluidity are split on whether or not team-member fluidity is detrimental to teams (Kerrissey et al., 2021). Our study, however, evidences a disconnect between team-member fluidity to both team coordination and team performance. Reasons for this disconnect may be due, in part, to characteristics of the primary care teams studied. As part of the PACT implementation, roles and responsibilities are clearly defined (Veterans Health Administration Department of Veteran Affairs, 2014), which may preserve shared mental models related to taskwork, coordination, and performance in the event of a team member change. Secondly, healthcare team member rotations are common (Bedwell et al., 2012; Tannenbaum et al., 2012), as team member changes may be strategic reallocation to balance workload or expertise. This may in turn make healthcare teams more adept at handling team member change over the time (Maynard et al., 2015; Kennedy et al., 2016; Gregory et al., 2021). This interpretation of the results should be considered with caution as teamwork processes which are critical to team adaptation and performance (e.g., coordination) should be bolstered periodically to avoid team breakdown (i.e., brittleness) over time.

### Implications

Our study leverages one of the largest, national datasets representative of US healthcare teams working in primary care. Our findings appear amidst a global crisis in the demand and capacity of healthcare personnel; namely, PCPs reported intention to reduce work hours and 20% intend to leave the profession within the next 2 years (Sinksy et al., 2021). The consequences of extended working shifts, increased volume and severity of patients, stress, anxiety, and fear have increased the rates of occupational burnout and mental health problems among frontline caregivers (Søvold et al., 2021). As such, our findings bear several practical implications for organizations, teams, and “essential workers”.

First, team-member fluidity in primary care teams was somewhat more likely within a team when the rate of COVID-19 was high and countermeasures to counteract the spread of COVID-19 were few. Although teamwork can buffer negative effects of adversity, higher team-member fluidity and staffing shortages can increase primary care team members’ workload and exacerbate pre-existing stress (Helfrich et al., 2017). This increase in workload places a burden on individuals and the team, prospectively breaking down teamwork as a result (Razinskas and Hoegl, 2020).

#### Bolstering teams through targeted team training

Beyond patient outcomes, adherence to a team-based approach translates to several benefits for health care workers, including higher job satisfaction and less burnout (Nelson et al., 2014). The continuous surge of information during the pandemic requires primary care teams to rapidly update their practices, demanding clinicians keep learning quickly about the disease, treatments, and preventative measures (Krist et al., 2020). Although a need remains to target countermeasures at the level of the intended area of reinforcement (e.g., organizational level interventions for organizational level needs), team training (i.e., training that targets the acquisition of knowledge, skills, and attitudes [KSAs] amenable to accomplishing shared goals) provides an effective countermeasure for applied teams (McEwan et al., 2017). Team training for team adaptation in healthcare with a focus on individual-level, generalizable teamwork KSAs (Hughes et al., 2016) is a warranted strategy to bolster teamwork KSAs (Bedwell et al., 2012; Gregory et al., 2021), and recommended (DeRue et al., 2008; Bedwell et al., 2012) for developing member-fluid teams. This training is not only necessary for applied teams working in adaptable, fluid contexts, but is severely lacking in the outpatient team-based care setting (Marlow et al., 2017; Fiscella and McDaniel, 2018). Considerations for specific team training needs in outpatient care are warranted.

### Limitations and future research

The current study, as with all research, has limitations. First, the study relies on archival COVID-19 data, which has known limitations (e.g., delays of COVID-19 infections being recorded accurately; Kamb, 2020). Secondly, two levels of analysis were employed. To test Hypothesis 3, data were aggregated to the station level, which may restrict our level 2 analytic power, which examines the impact of adversity (i.e., COVID-19 rates) on various outcomes. Additionally, our study examined team-member fluidity in the context of team turnover, which was dichotomized as a team member leaving the team. This operationalization failed to account for introducing a new team member, which may have effects on adaptation and resilience through the addition of team resources. As past research established a negative association between team-member fluidity and team cognitions (Bedwell, 2019), future work should examine the structures driving team-member fluidity (e.g., turnover vs. reassignment) and the impact of team-member role rotation on overall team adaptation (e.g., turnover of clerks vs. PCPs; DeRue et al., 2008; Tannenbaum et al., 2012; Fiscella and McDaniel, 2018). Of note, the study’s second hypothesis and associated outlier reveal limitations in the study’s approach. Namely, our study sought to understand extreme teams working within this novel context of pandemic stress. Our study includes a significant outlier; however, inclusion of the VHA center in New York at the height of the pandemic’s first wave was deemed as integral to answering the study questions. Additionally, our study used proxy measures for team coordination and performance, as guided by health services research (Crawford et al., 2019) and behavioral marker definitions of team coordination (Salas et al., 2008). While we found that coordination significantly predicted team performance, this relationship may be an artifact of the measurements (i.e., management of BP could predict ER utilization). Lastly, hypothesized mediating effects specified predictions which may have complicated the interpretation of indirect effects (i.e., effects would have canceled each other out). These relationships were specified in accordance with the science of team resilience in applied settings, with an emphasis on examining ‘teams in the wild.’ Applying the latest advancements in teamwork science led to the development of the model, allowing for exploration of the hypothesized relationships in applied extreme team adaptations.

### A call for greater conceptual clarity

Notably, the current investigation applies teamwork theories to applied primary care teams working during initial waves of the COVID-19 pandemic. The approach necessitated a transdisciplinary examination of healthcare “teams in the wild: – merging perspectives of team resilience and coordination from teamwork science with coordination of care literature in health services research. Understandably, our study limitations highlight gaps in the field, particularly for applied teams research. As such, we have dedicated this segment for calls to improve clarity in critical concepts. First is the need to continue clarifying concepts underlying team resilience. Currently, team resilience can be conceptualized as a process or outcome and is thought to be the by-product of interactions among the team over time. Emergence (of team concepts) – by nature – is often multi-level (Coultas et al., 2014). Yet, patterns of emergence for measuring adversity’s likely effects on teams are nascent at best (see Dietz et al., 2017 for role of individual factors in team stress response) with considerable conceptual overlap with similar areas of teams research. For example, the current study examined ‘team member fluidity’ as a trigger to team adaptation in the context of resilience. Yet, changes in team membership are listed in seminal reviews as a trigger to team ‘stress’ (Dietz et al., 2017), ‘adaptation’ (Maynard et al., 2015), and ‘resilience’ (Alliger et al., 2015), complicating cohesive research efforts. Secondly, research on teamwork in healthcare is gaining momentum (Dinh et al., 2020). Yet, despite teamwork’s clear involvement in clinical performance (Schmutz et al., 2019) and patient-relevant outcomes (Hughes et al., 2016), there is a disconnect between best practices of teams research (e.g., preference for psychometrically robust measures which require additional data collection) and the reality of healthcare service delivery (e.g., strong preference for feasible behavioral measures ideally linked to measures already captured at point-of-care). To bridge this gap, we leveraged the definition of coordination in behavioral markers (Salas et al., 2008) and prior research on team performance (Crawford et al., 2019) to identify BP measurement and ER utilization as proxy measures for coordination and performance. However, future work should further distinguish concepts of “coordination” – as teamwork process(es) – and “care coordination” – which may often be more oriented toward team taskwork processes and performance.

## Conclusion

Advancing and testing a theoretical model of team resilience in the presence of adversity yields several key insights on how teams function in applied settings. First, coordination plays a key role in team performance. Interestingly, team-member fluidity had little to no impact on coordination or performance. Second, intensity of the adversity alone did not predict team-member fluidity; however, team-member fluidity was more likely to occur in areas with an intense stimulus and low presence of countermeasures. Future work should examine the conditions under which organizational adaptation may ideally occur and further investigate predictors of team coordination in the presence of an adverse stimulus. Additionally, future research should examine how the turnover of each specific role within the team (e.g., clerk or nurse) impacts team performance.

## Data availability statement

The data analyzed in this study is subject to the following licenses/restrictions: data obtained for the purposes of this study are a combination of publicly available datasets and data available through the Veterans Health Administration’s Corporate Data Warehouse. Requests to access these datasets should be directed to hysong@bcm.edu.

## Ethics statement

The study was reviewed and approved by Baylor College of Medicine Institutional Review Board (protocol # H-42358). The study was conducted in accordance with the local legislation and institutional requirements. The ethics committee/institutional review board waived the requirement of written informed consent for participation from the participants or the participants’ legal guardians/next of kin because consent to participate and consent to publish are not applicable for this study—this is a database review of team-level data and no individual information will be reported.

## Author contributions

AH: Funding acquisition, Supervision, Visualization, Writing – original draft, Writing – review & editing, Conceptualization. KA: Conceptualization, Investigation, Supervision, Visualization, Writing – original draft. HL: Data curation, Formal analysis, Methodology, Software, Writing – review & editing. FO: Data curation, Funding acquisition, Investigation, Methodology, Supervision, Writing – review & editing. TP: Data curation, Investigation, Project administration, Validation, Visualization, Writing – original draft, Writing – review & editing. CJ: Data curation, Methodology, Project administration, Resources, Software, Writing – review & editing. SH: Conceptualization, Funding acquisition, Investigation, Resources, Supervision, Writing – review & editing.

## Footnotes

^1^We interpreted the results here as if larger values indicate better performance. However, that is not the case for the team performance metric. The results in the Tables are presented in the original (i.e., lower is better metric).
