# Supplementary material for: What can we learn from COVID-19?: examining the resilience of primary care teams
Source: Front Psychol. 2023 Nov 22;14:1265529. doi: 10.3389/fpsyg.2023.1265529 (PMC10703302; doi:10.3389/fpsyg.2023.1265529)
Supplement: Supplementary file 1 [file Data_Sheet_1.docx]

**Appendix A**

This appendix contains the Bayesian details of our analytic models. Trace plots, histograms of the posterior distribution, and parameter estimate autocorrelation plots are presented for every parameter in the model. This process is repeated for each hypothesis with/without the outlier clinic.

Data were analyzed using Mplus version 8.3 software program (Muthén, 2012) using Gibbs sampling (Yuan & MacKinnon, 2009). Specifically, we used set seed values (including 985, 5151, and 1989), and ran 4 Markov Chain Monte Carlo (MCMC) chains for each parameter, using default starting values. Convergence was assessed using the potential scale reduction factor convergence diagnostic, and model fit was assessed using the posterior predictive p value. The first half of each chain was discarded as initial burn-in. The minimum and maximum number of iterations were set to 10,000 and 100,000, respectively. The MCMC chains were both statistically tested and visually inspected (trace plots) to ensure that the posterior distribution of the model parameters reached convergence. We used default Mplus prior settings for all parameters, intended to not influence posterior distributions. Teams were specified as the lowest level of analysis, nested within facilities, aggregated to the station level. All outcomes variables except for team-member fluidity were treated as conditionally normally distributed in our analytic model. Team-member fluidity was dichotomized to reflect whether a team-member left a team within a given month.

**Hypothesis 1 with Outlier**

**Trace Plots**

Trace plots display the sampled estimates for each iteration of the Markov Chain Monte Carlo process. Ideally the plots look like random noise with a slope of zero.

**Figure A1**

*Trace plot for level-one residual variance for Team Performance*


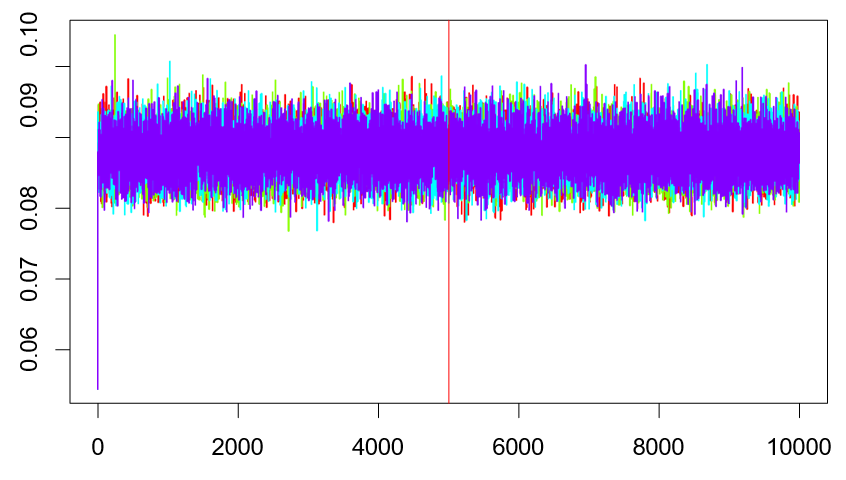


**Figure A2**

*Trace plot for the fixed intercept for Team Performance*


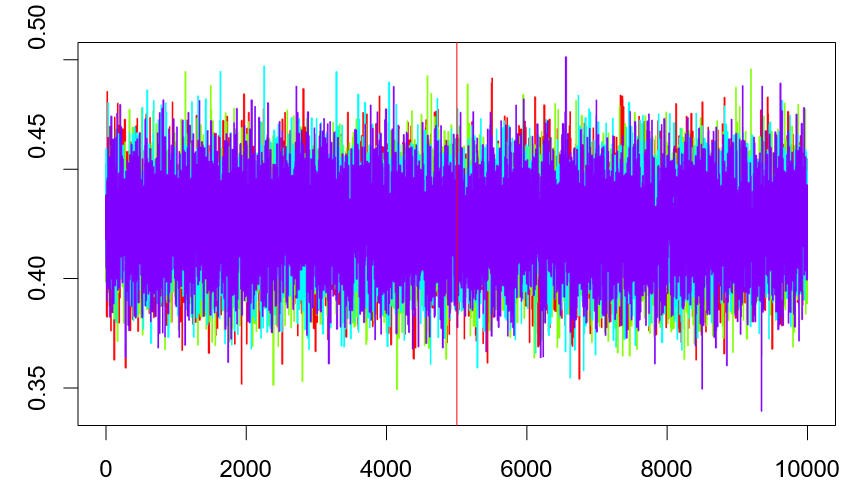


**Figure A3**

*Trace plot of Level 2 Relationship: Team Performance ON Adversity*


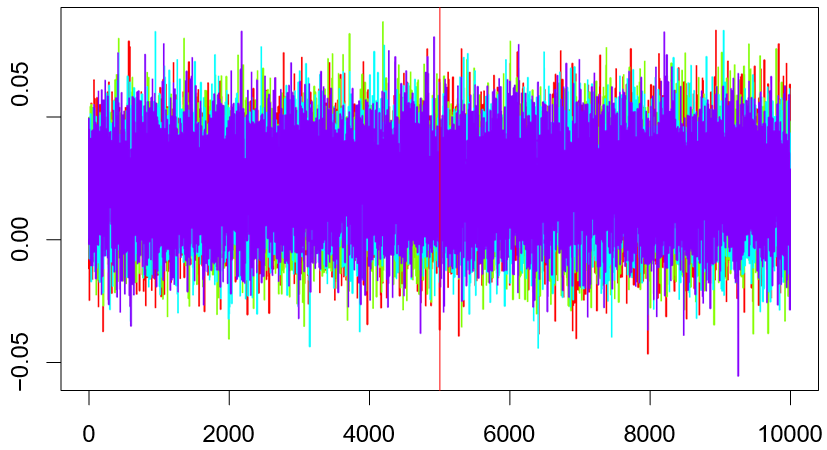


**Figure A4**

*Trace plot of Level 2 Residual for Team Performance*


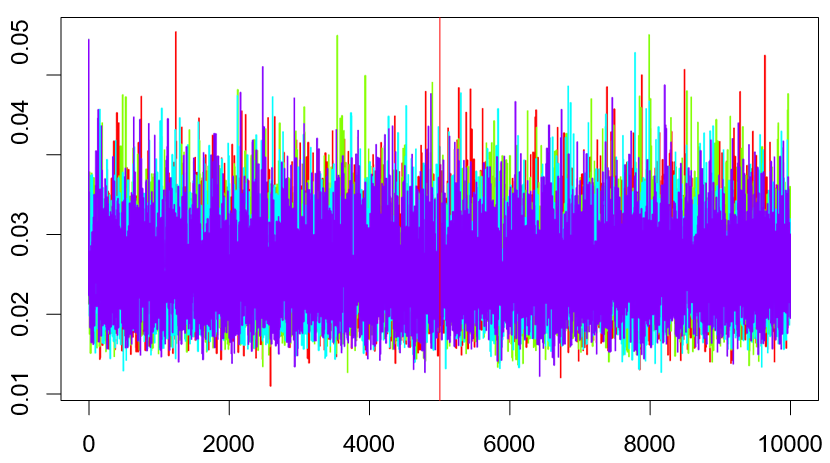


**Posterior Distributions for the Parameters with Outlier**

Posterior distributions reflect the updated plausibility of the parameter values after the likelihood has been combined with the prior distribution.

**Figure A5**

*Posterior Distribution of level-one residual variance for Team Performance*


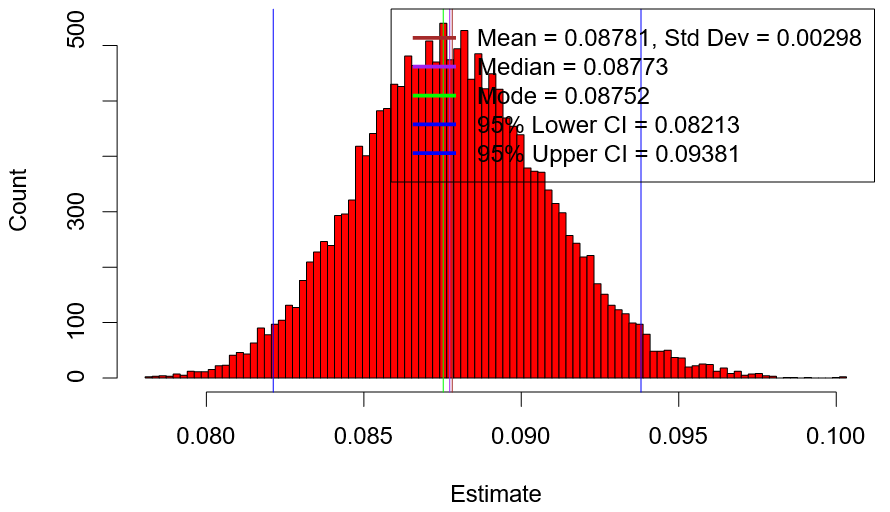


**Figure A6**

*Posterior Distribution of the fixed intercept for Team Performance*


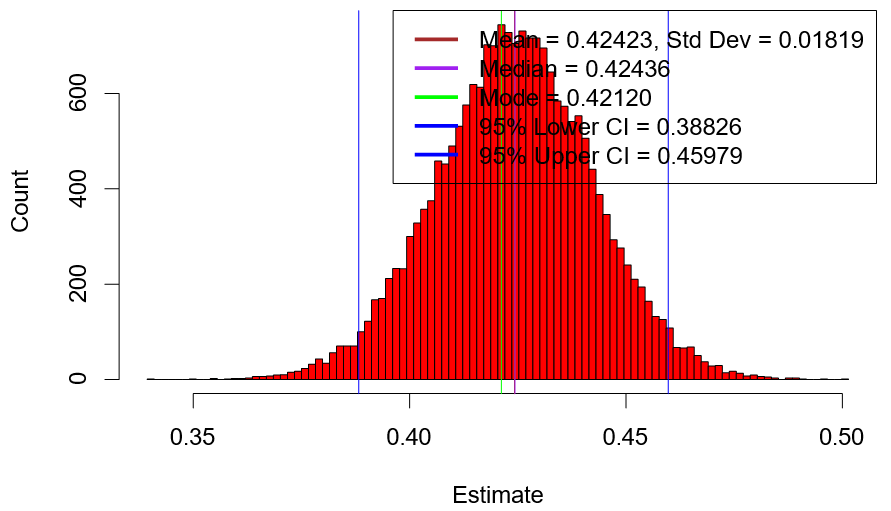


**Figure A7**

*Posterior Distribution of the Level 2 Relationship: Team Performance ON Adversity*


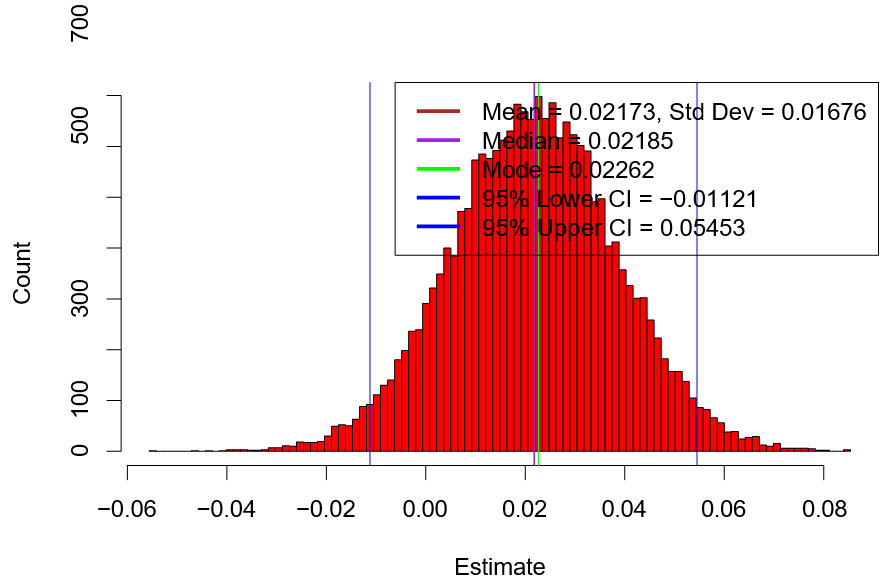


**Figure A8**

*Posterior Distribution for Level-two Residual Variance for Adaptive Team Performance*


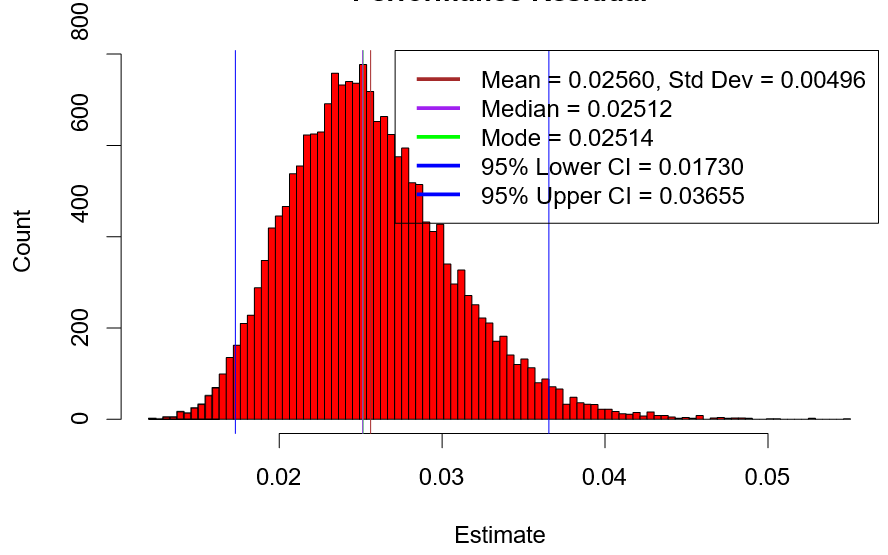


**Autocorrelation Plots with Outlier**

Autocorrelation plots represent a chain’s correlation at successive lags. High correlations indicate the need for more samples. All plots provided in this appendix are from the se

**Figure A9**

*Autocorrelation plot for level-one residual variance for Team Performance*


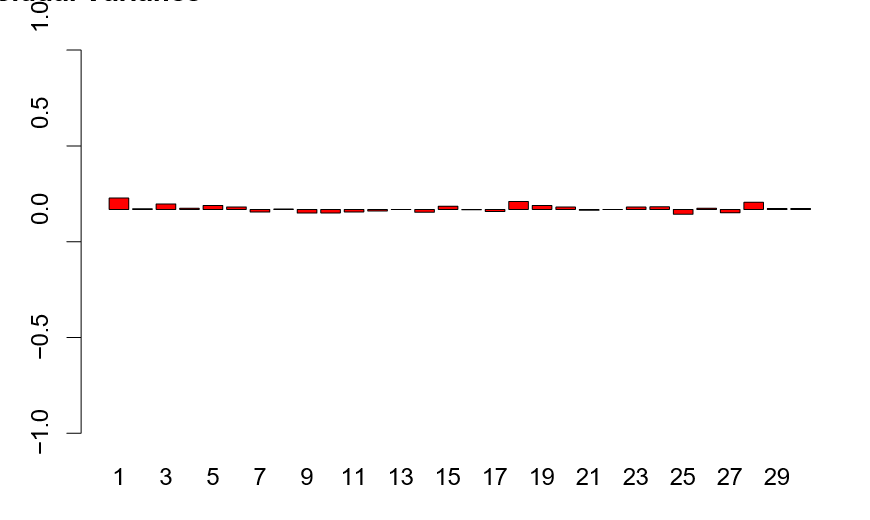


**Figure A10**

*Autocorrelation plot for the fixed intercept for Team Performance*


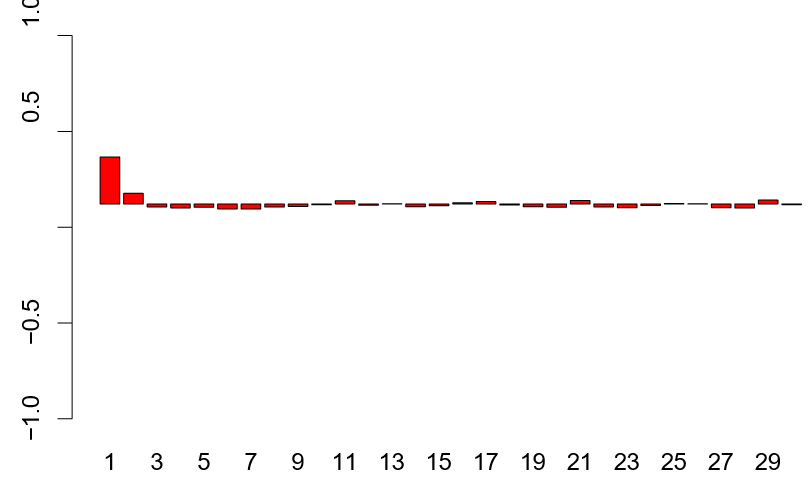


**Figure A11**

*Autocorrelation plot of Level 2 Relationship: Team Performance ON Adversity*


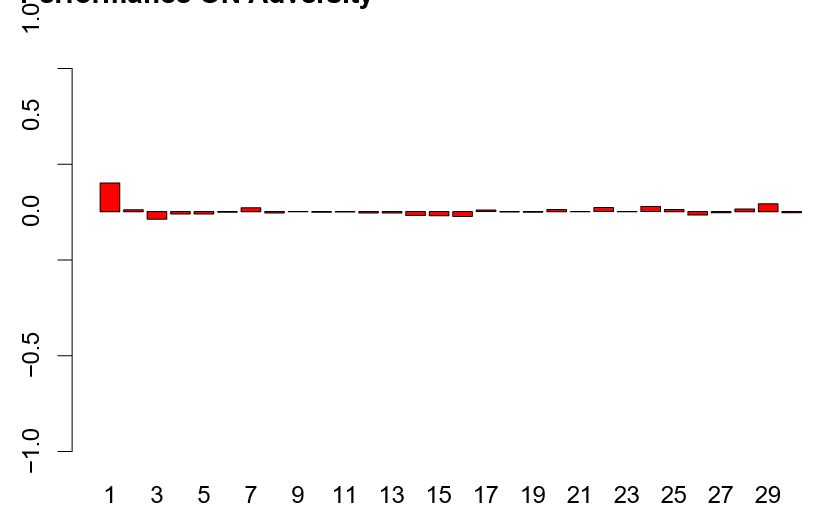


**Figure A12**

*Autocorrelation plot for Level-two Residual Variance for Team Performance*


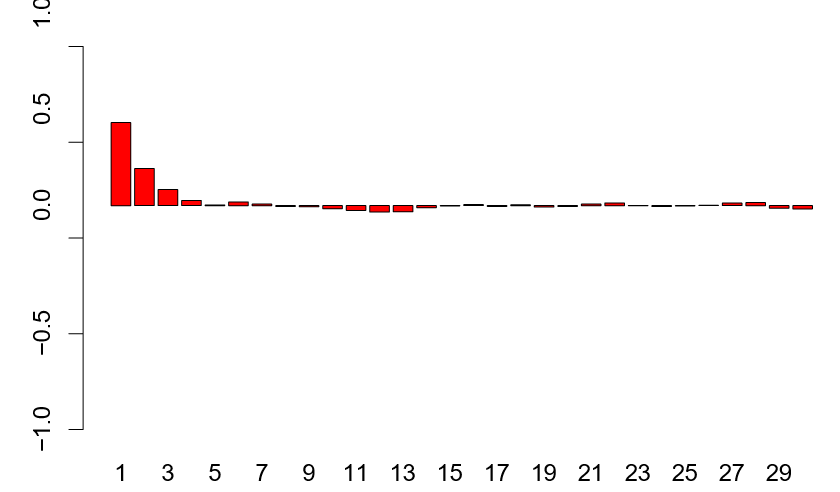


**Hypotheseis 1 Without Outlier**

**Trace Plots without Outlier**

**Figure A13**

*Trace plot for level-one residual variance for Team Performance*


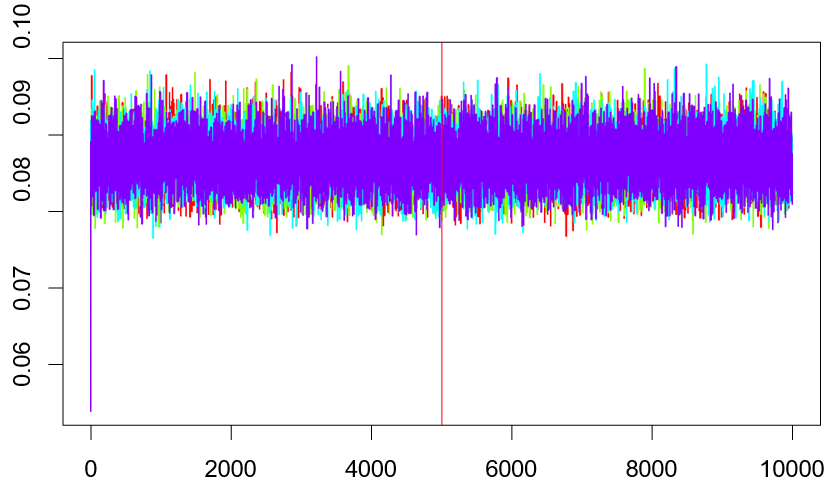


**Figure A14**

*Trace plot for the fixed intercept for Team Performance*


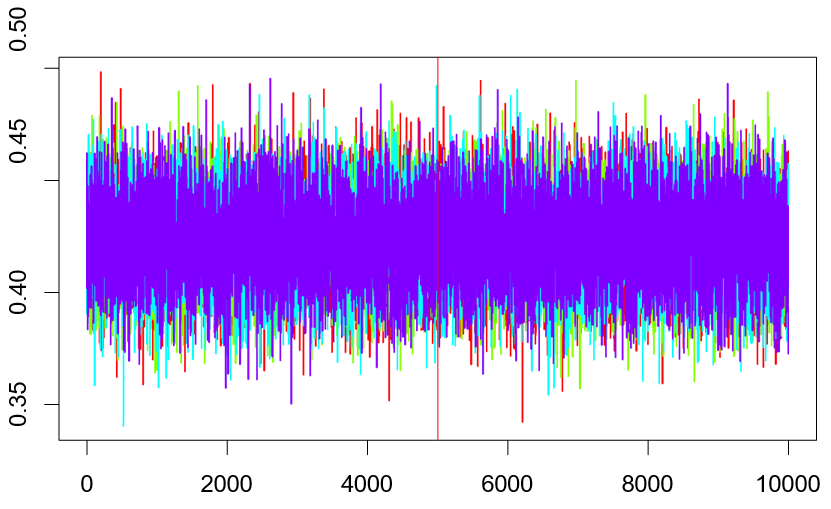


**Figure A15**

*Trace plot of Level 2 Relationship: Team Performance ON Adversity*


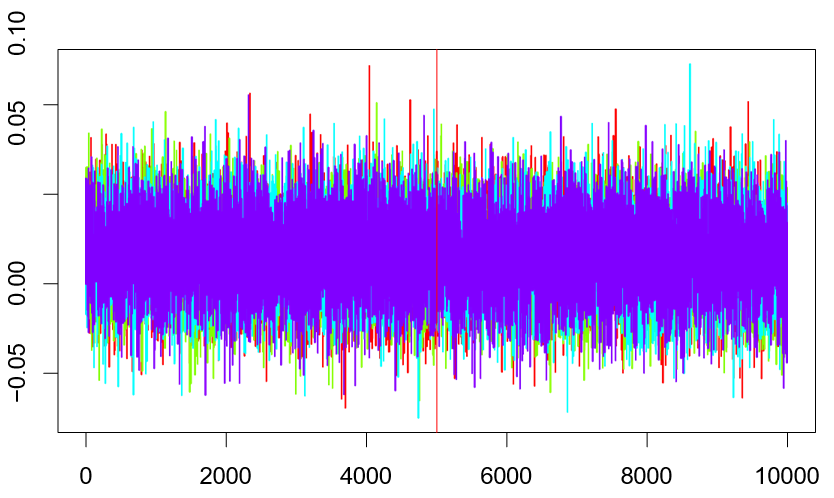


**Figure A16**

*Trace plot for level-two residual variance for Team Performance*


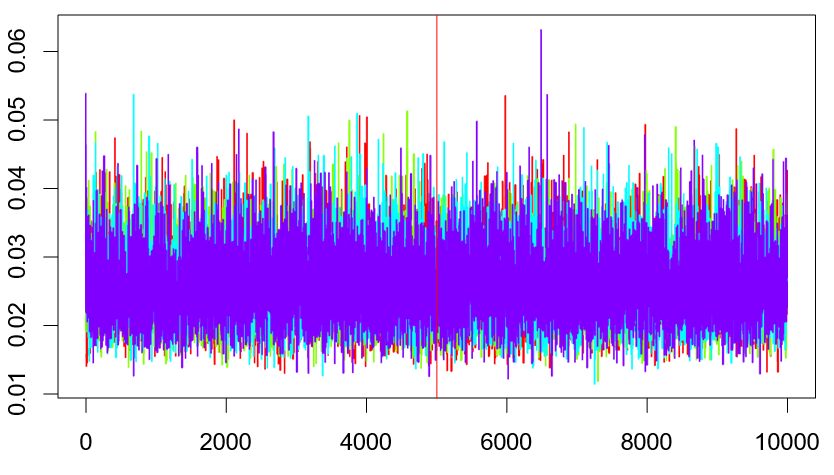


**Posterior Distributions for the Parameters without Outliers**

**Figure A17**

*Posterior Distribution of level-one residual variance for Team Performance*


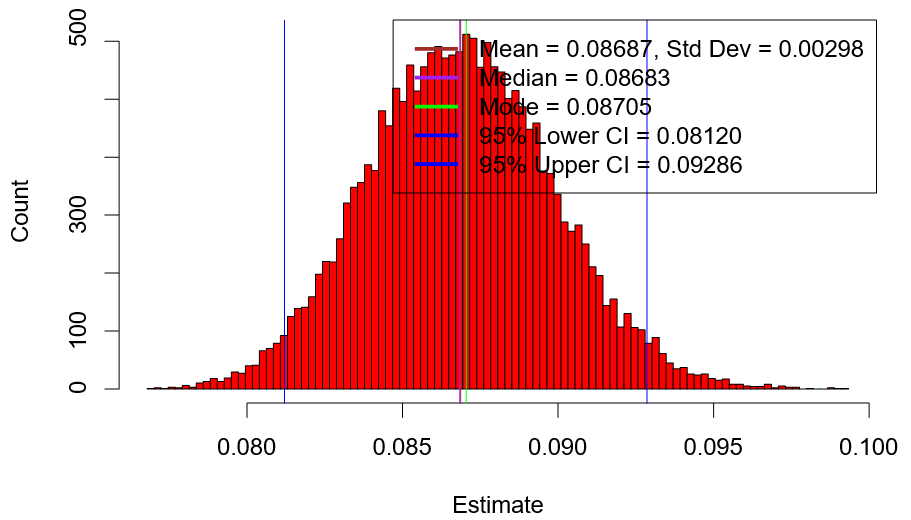


**Figure A18**

*Posterior Distribution of the fixed intercept for Team Performance*


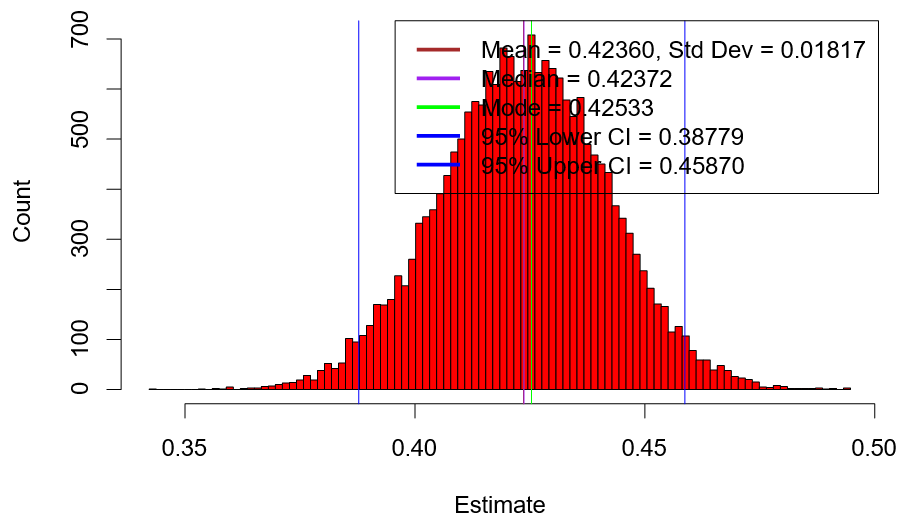


**Figure A19**

*Posterior Distribution of the Level 2 Relationship: Team Performance ON Adversity*


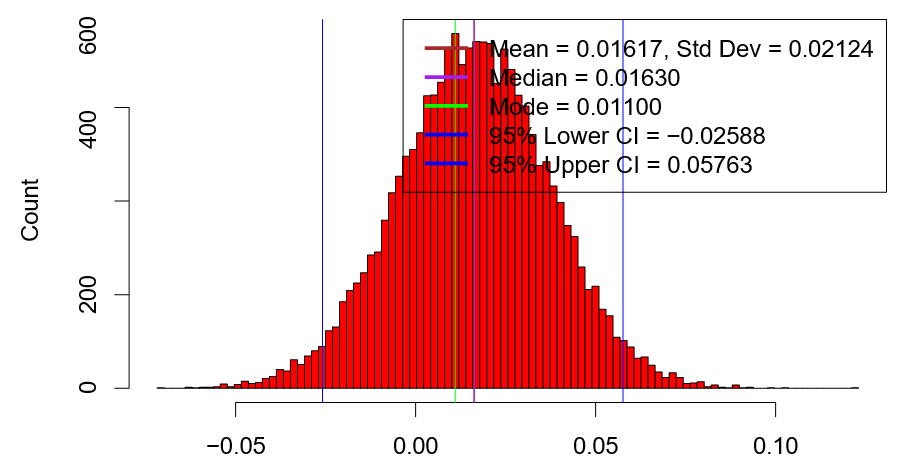


**Figure A20**

*Posterior Distribution for Level-two Residual Variance for Team Performance*


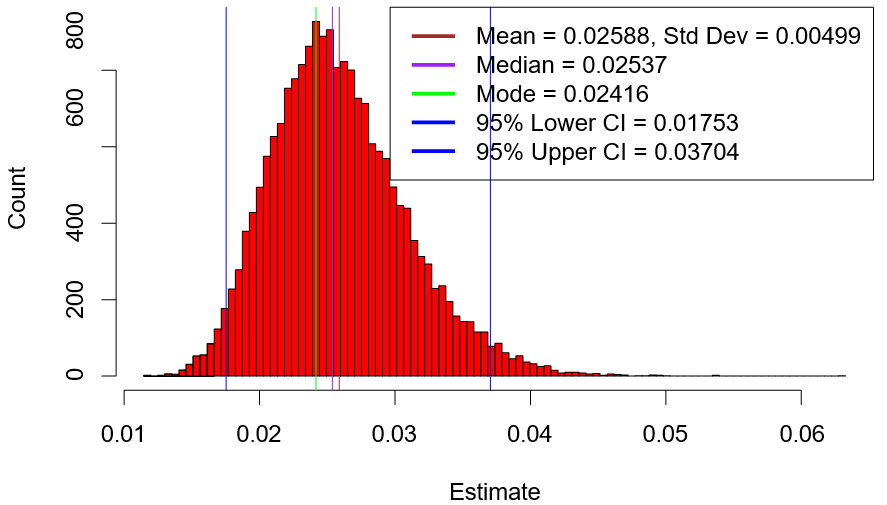


**Autocorrelation Plots without Outlier**

**Figure A21**

*Autocorrelation plot for level-one residual variance for Team Performance*


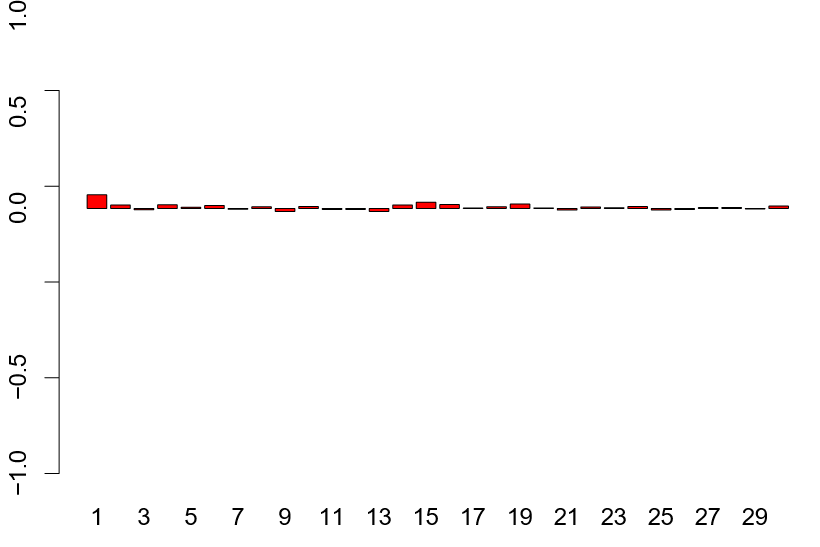


**Figure A22**

*Autocorrelation plot for the fixed intercept for Team Performance*


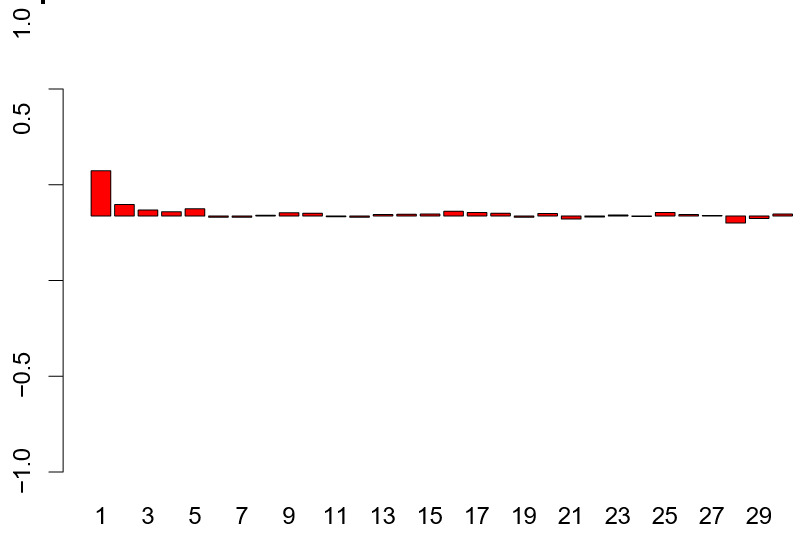


**Figure A23**

*Autocorrelation plot of Level 2 Relationship: Team Performance ON Adversity*


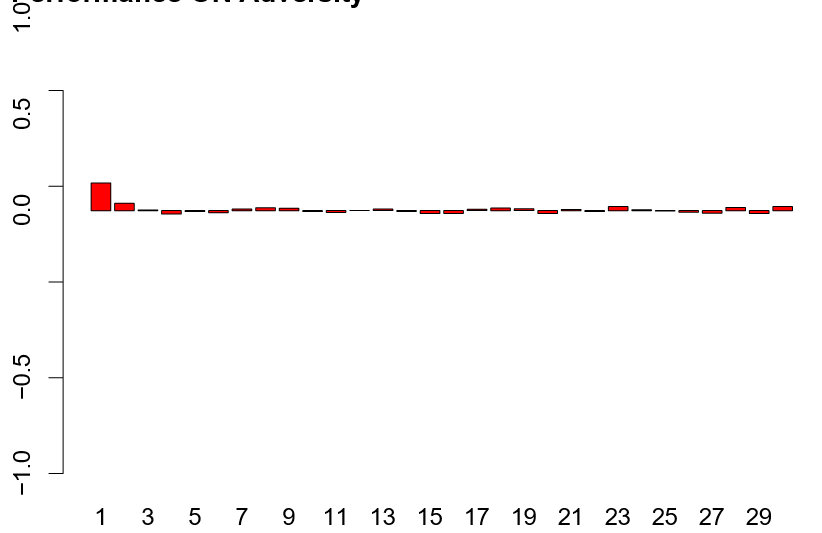


**Figure A26**

*Autocorrelation plot for Level-two Residual Variance for Team Performance*


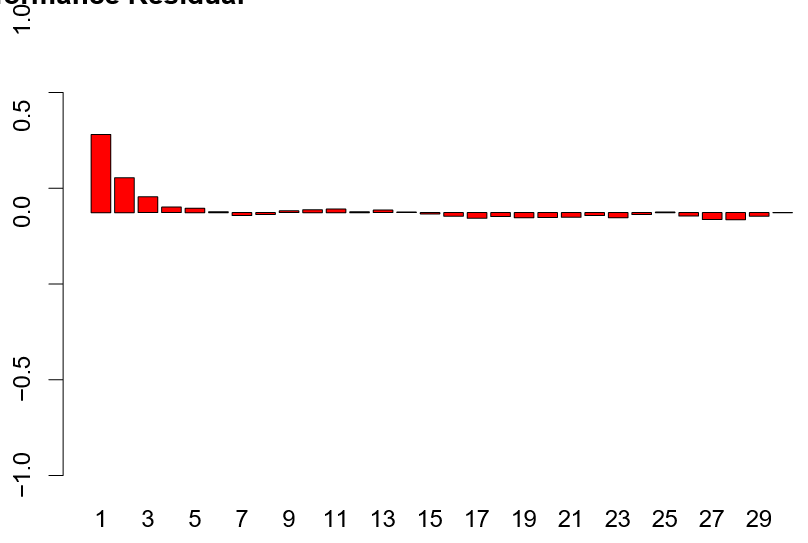


**Hypotheses 2 and 3 with Outlier**

**Trace Plots with Outlier**

**Figure A25**

*Trace Plot for Level-one Relationship of Team Coordination on Team Member Fluidity*

*
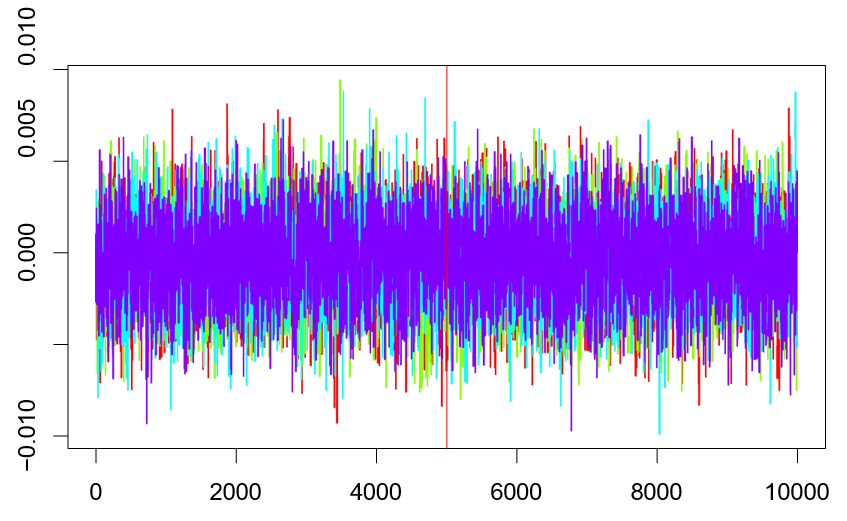
*

**Figure A26**

*Trace Plot for Level-one Relationship of Team Performance on Team Coordination*


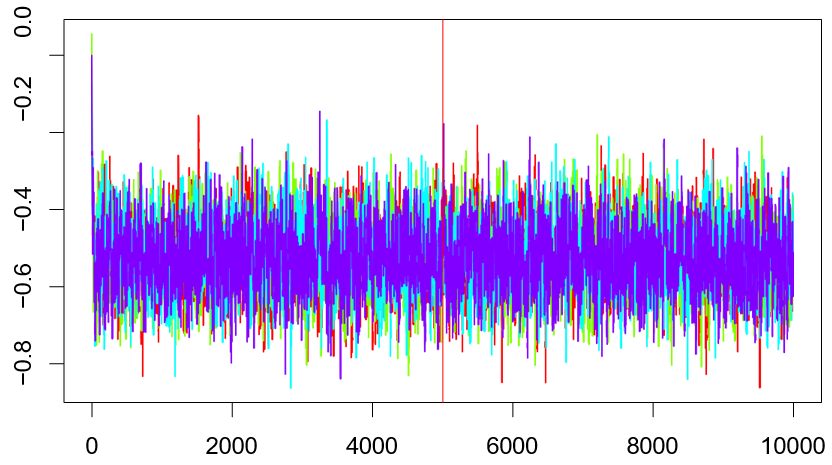


**Figure A27**

*Trace Plot for Level-one Variance for Team Coordination*


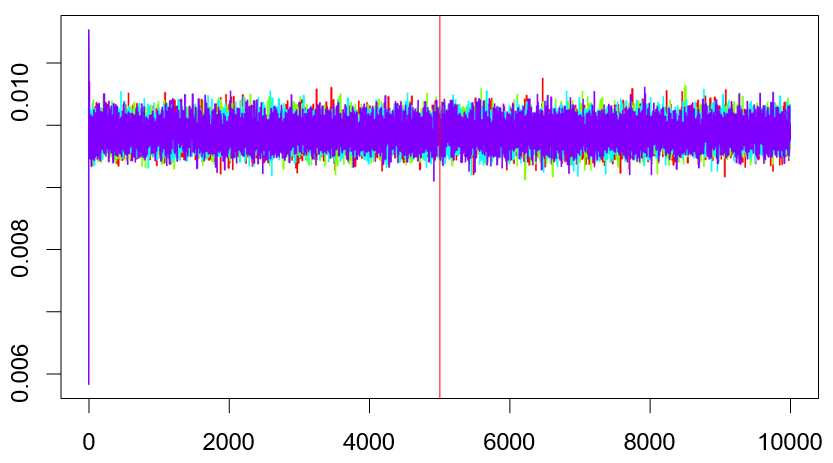


**Figure A28**

*Trace plot for level-one residual variance for Team Performance*


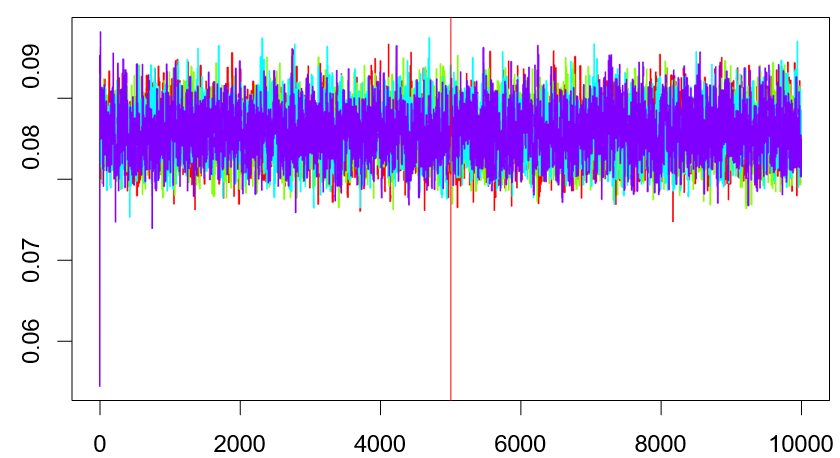


**Figure A29**

*Trace Plot for the Fixed Intercept for Team Coordination*


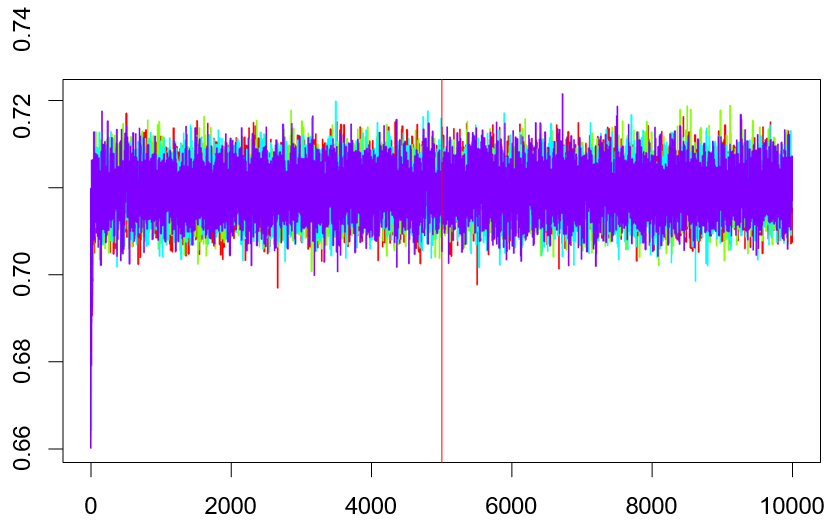


**Figure A30**

*Trace plot for the fixed intercept for Team Performance*


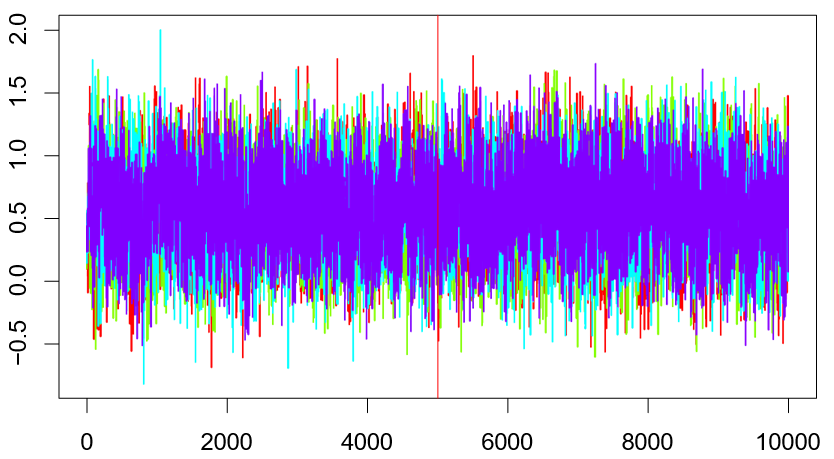


**Figure A31**

*Trace plot of Level 2 Relationship: Team member fluidity ON Adversity*


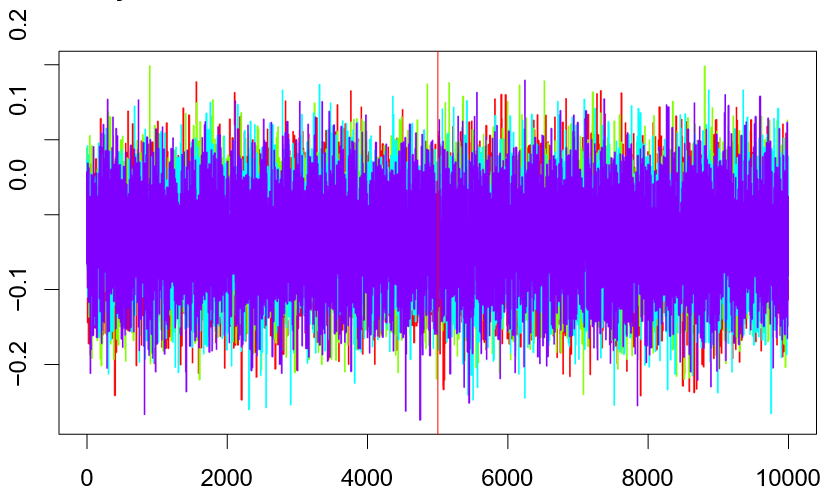


**Figure A32**

*Trace plot of Level 2 Relationship: Team member fluidity ON Countermeasures*


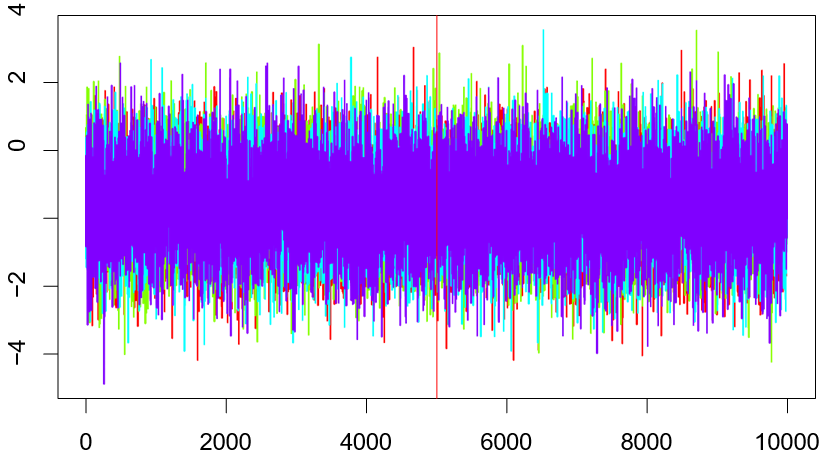


**Figure A33**

Trace plot of Level 2 Relationship: Team Coordination ON Team Member Fluidity


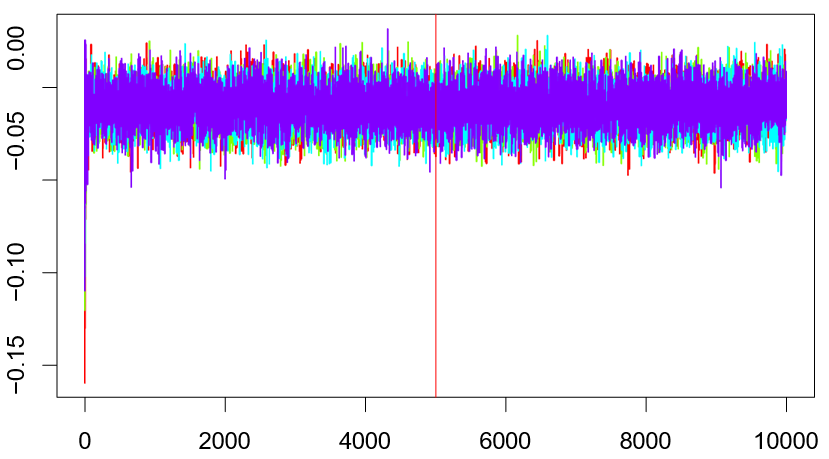


**Figure A34**

*Trace plot of Level 2 Relationship: Team performance ON Team coordination*


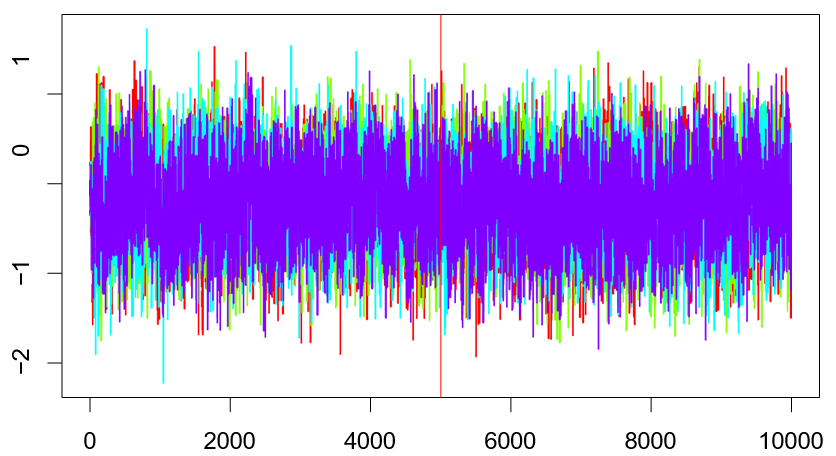


**Figure A35**

*Trace plot of Level 2 Relationship: Team Performance ON Adversity*


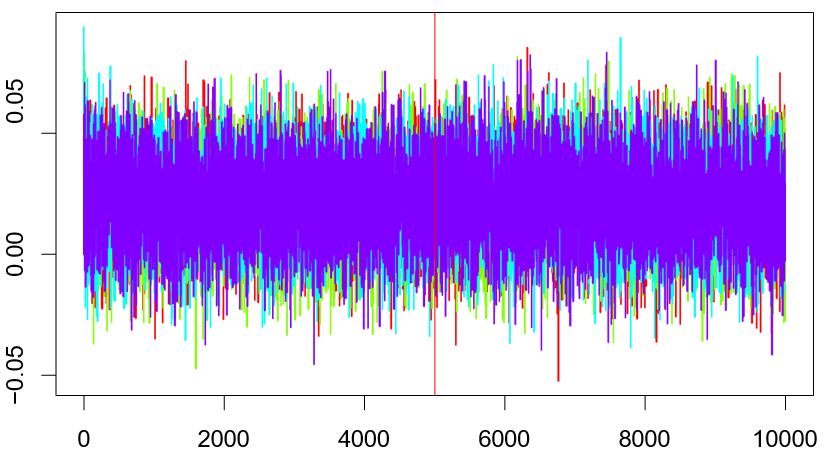


**Figure A36**

*Trace plot of Level 2 Relationship: Team Performance ON Countermeasure*


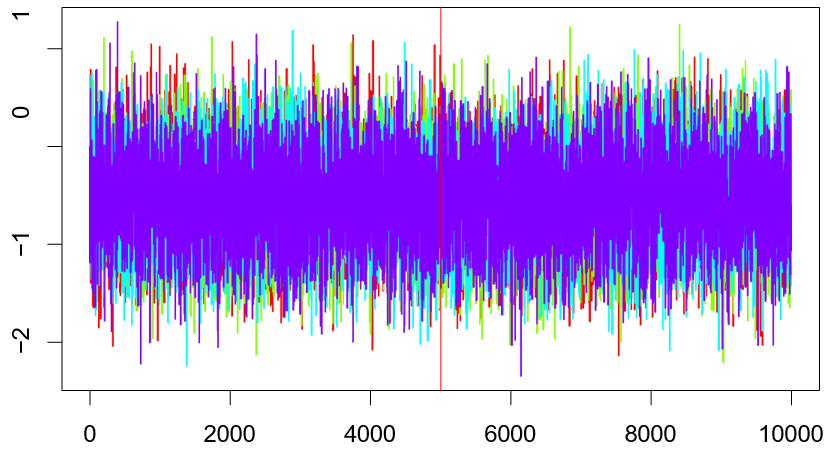


**Figure A37**

*Trace plot for level-two residual variance for Team member fluidity*


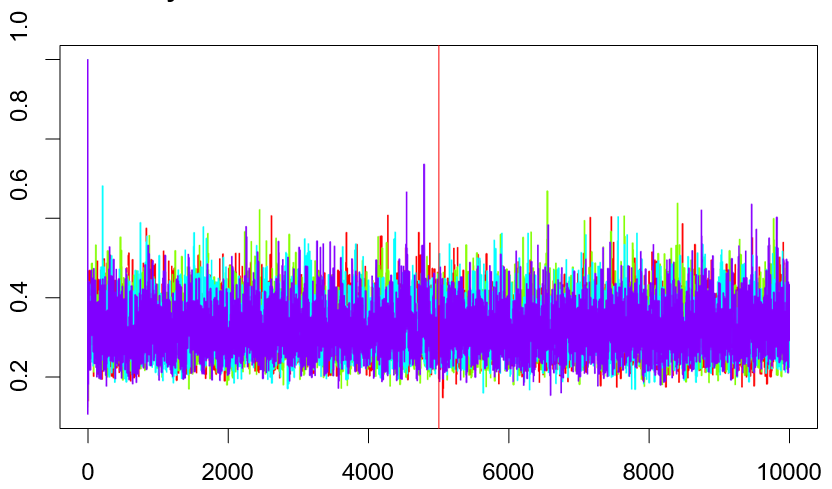


**Figure A38**

*Trace plot for level-two residual variance for Team Coordination*


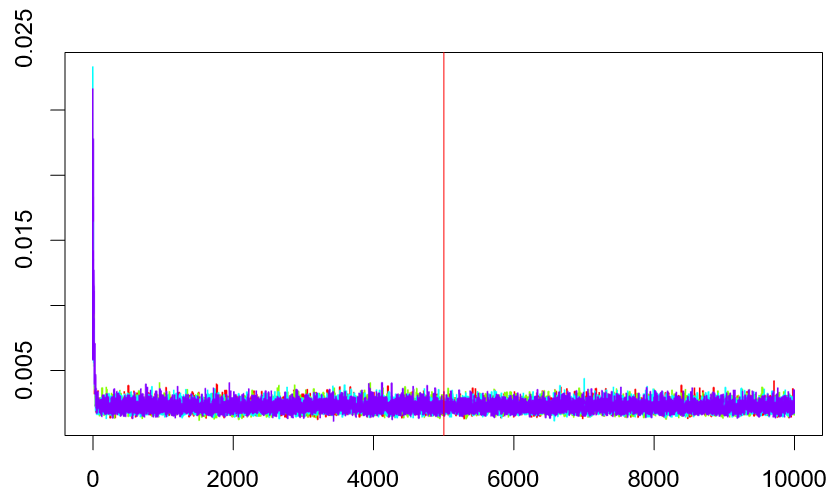


**Figure A39**

*Trace plot for level-two residual variance for Team performance*


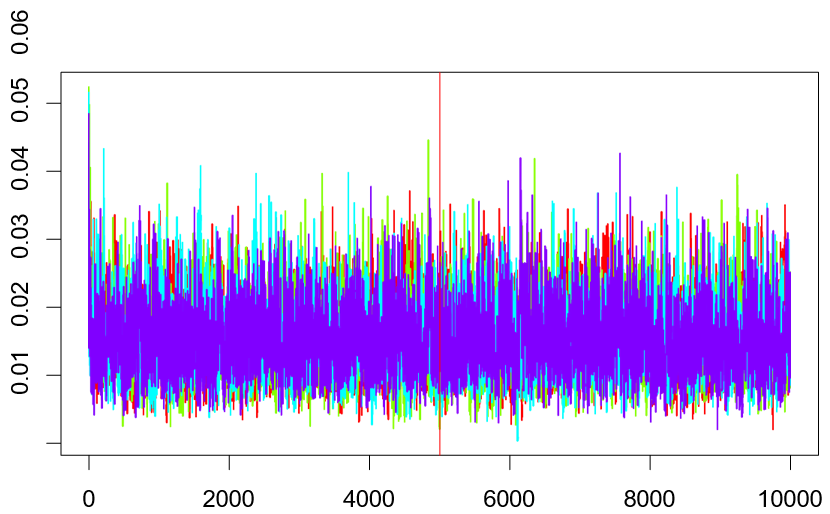


**Figure A40**

*Trace plot for the team member fluidity threshold*


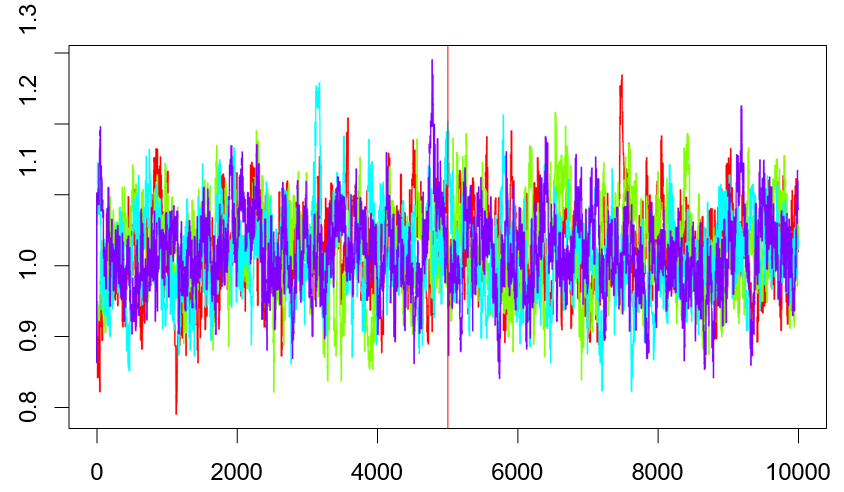


**Figure A41**

*Trace plot for the indirect effect of Adversity to Team performance*

*
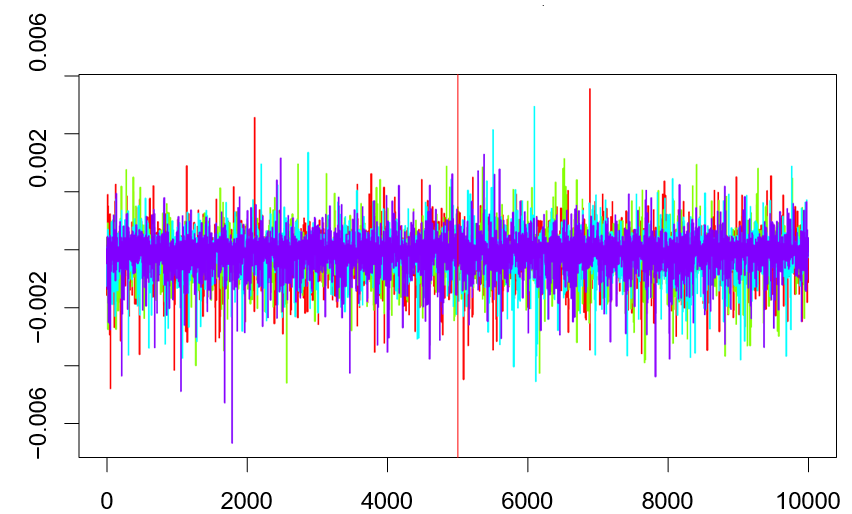
*

**Posterior Distributions for the Parameters with Outlier**

**Figure A42**

*Posterior Distribution for Level-one Relationship of Team Coordination on Team Member Fluidity*


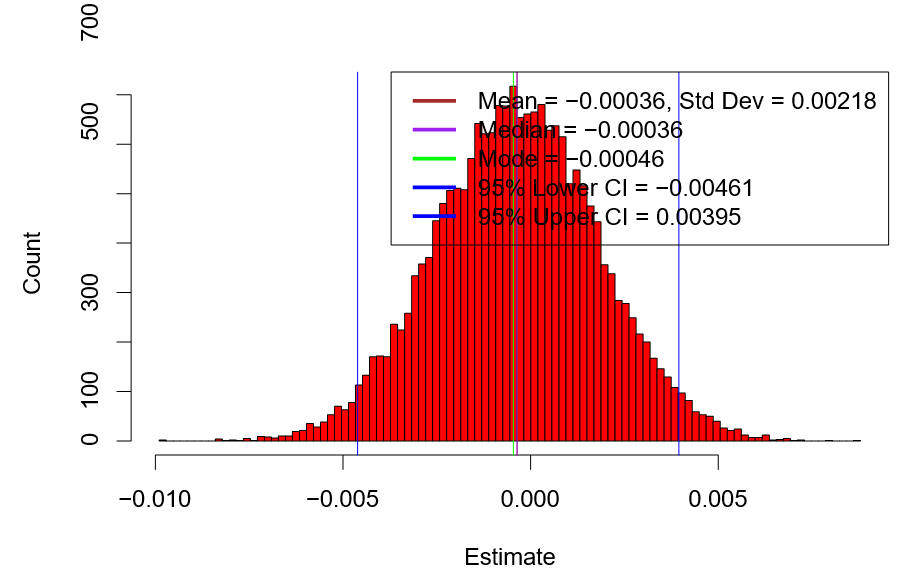


**Figure A43**

*Posterior Distribution for Level-one Relationship of Adaptive Team Performance on Team Coordination*


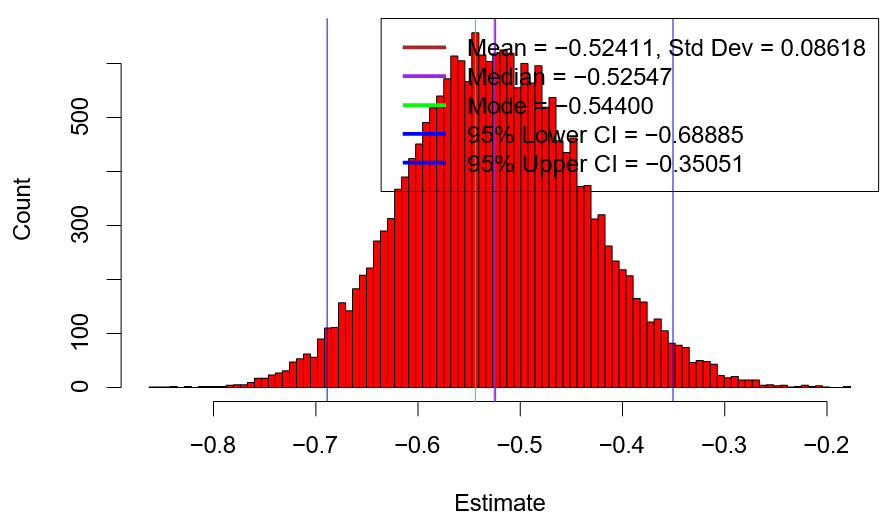


**Figure A44**

*Posterior Distribution for Level-one Residual Variance for Team Coordination*

500


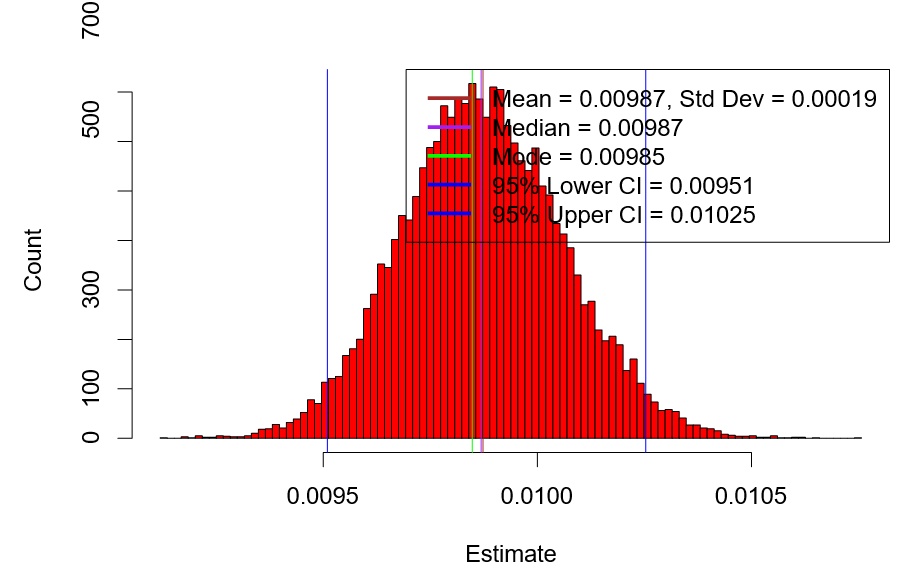


**Figure A45**

*Posterior Distribution for level-one variance for Team Performance*


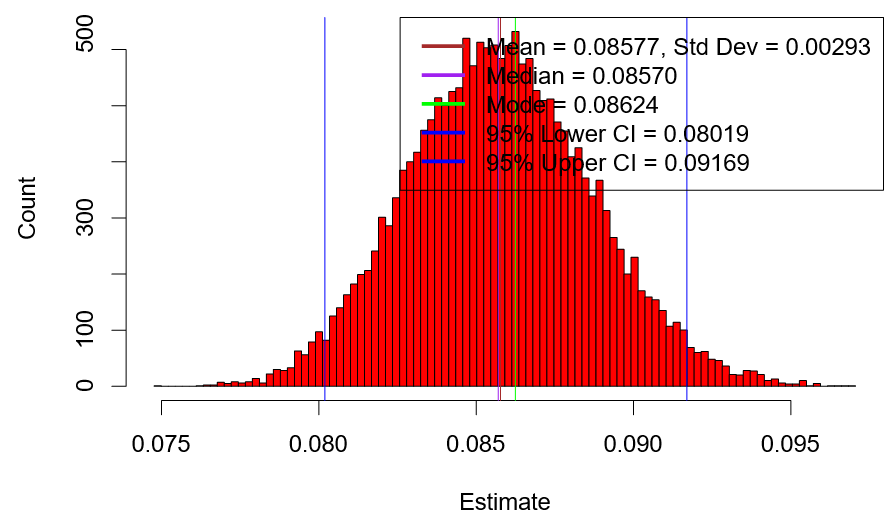


**Figure A46**

*Posterior Distribution for the Fixed Intercept for Team Coordination*


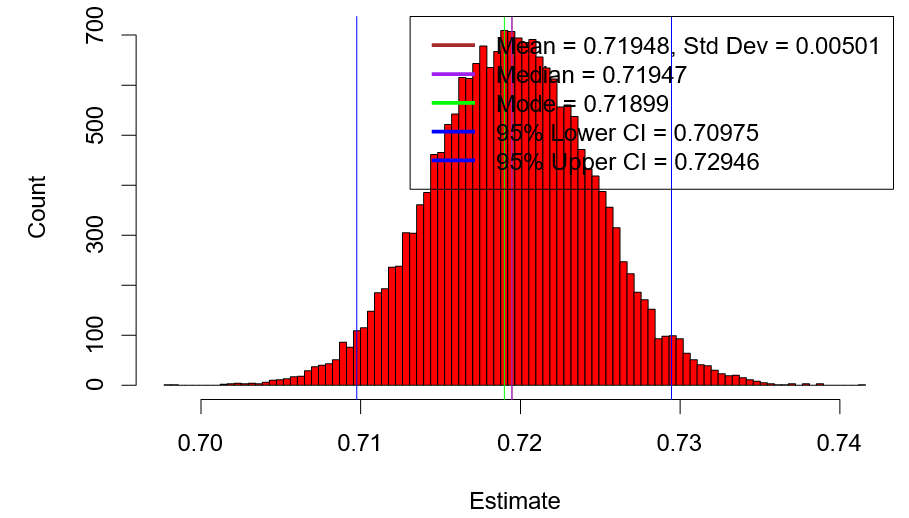


**Figure A 47**

*Posterior Distribution for the fixed intercept for Team Performance*


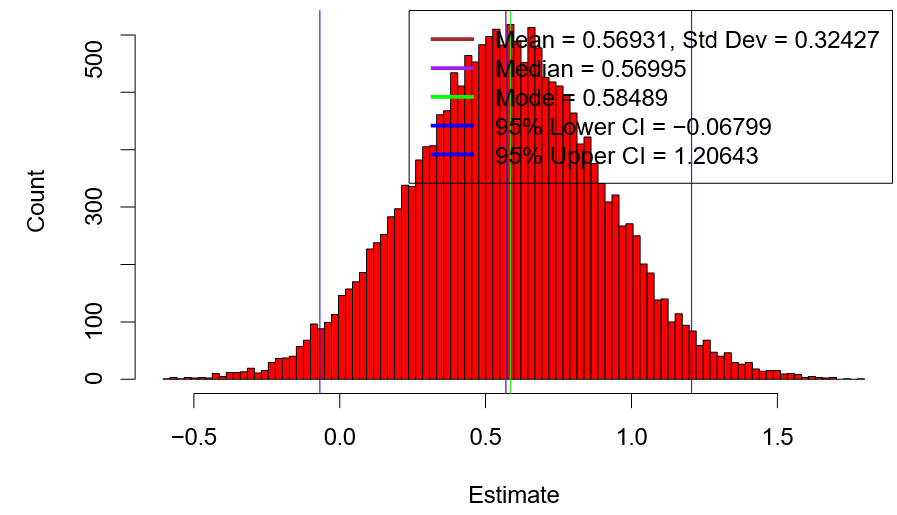


**Figure A48**

*Posterior Distribution of Level 2 Relationship: Team member fluidity ON Adversity*


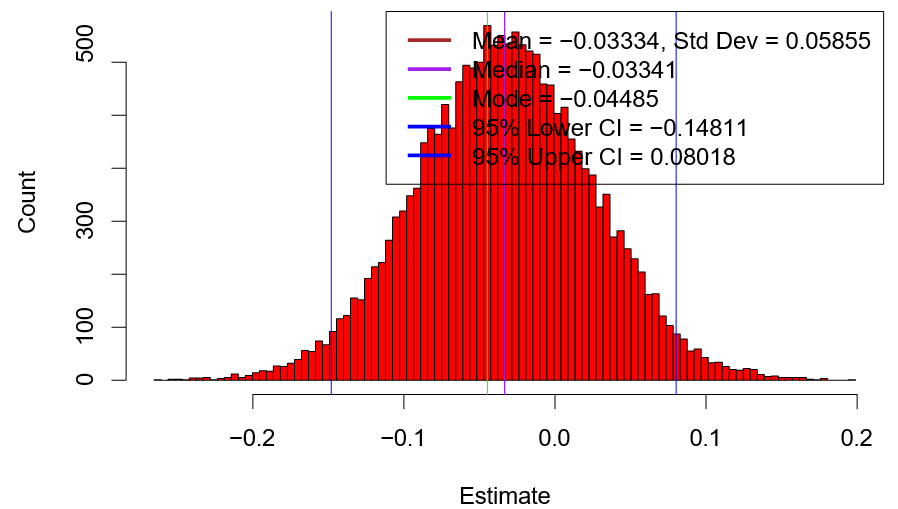


**Figure A49**

*Posterior Distribution of Level 2 Relationship: Team member fluidity ON Countermeasures*


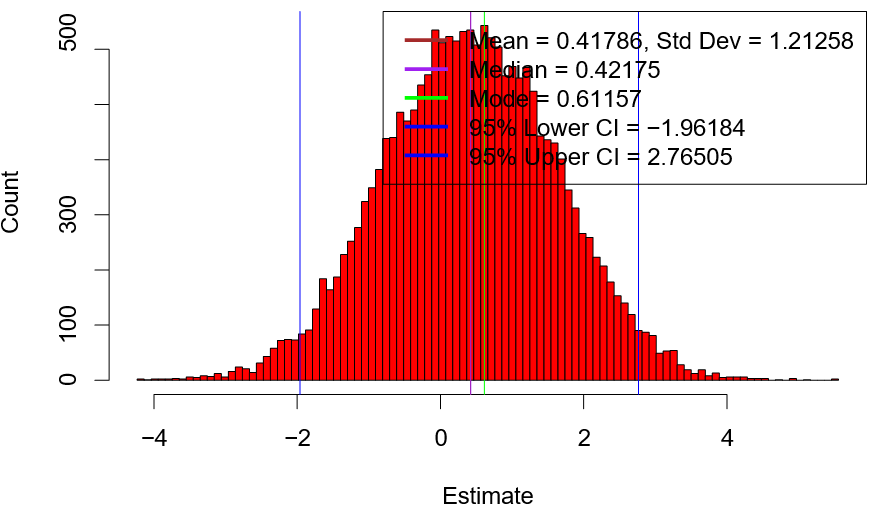


**Figure A50**

*Posterior Distribution of Level 2 Relationship: Team coordination ON team member fluidity*


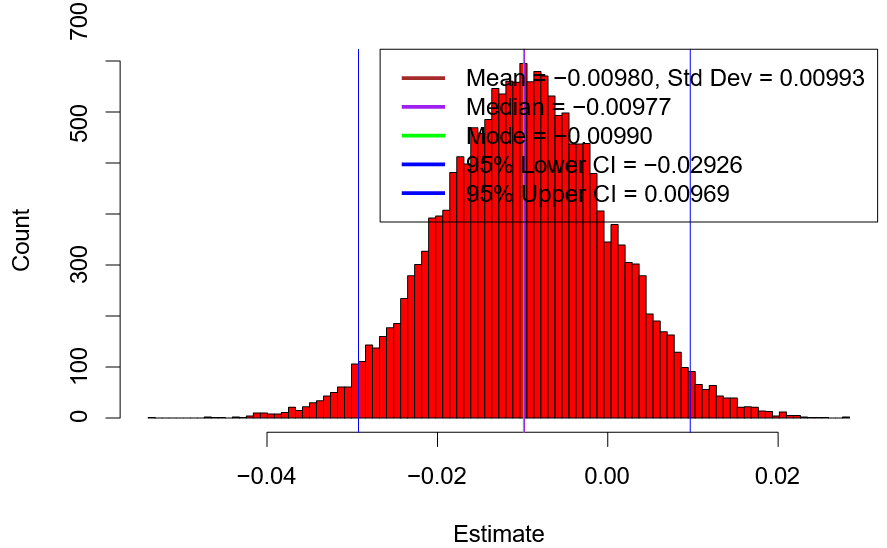


**Figure A51**

*Posterior Distribution of Level 2 Relationship: Team Performance ON Team Coordination*


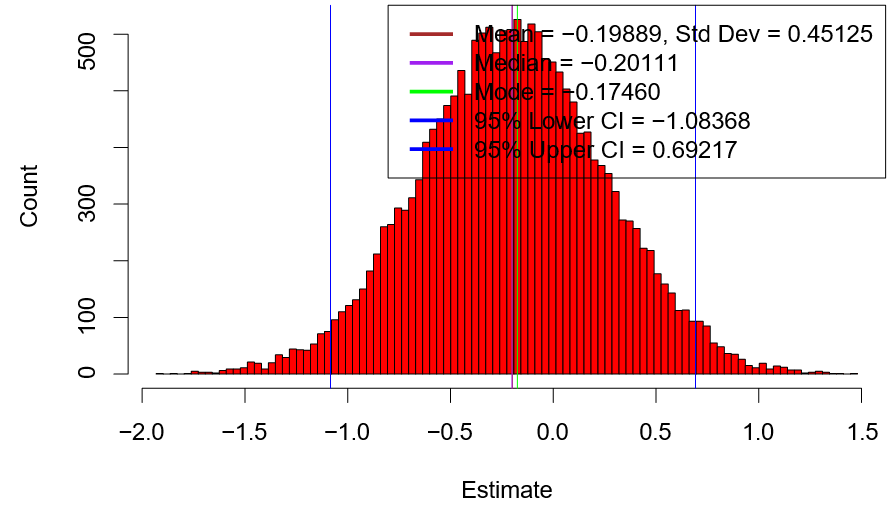


## Figure A52

*Posterior Distribution of Level 2 Relationship: Team Performance ON Adversity*


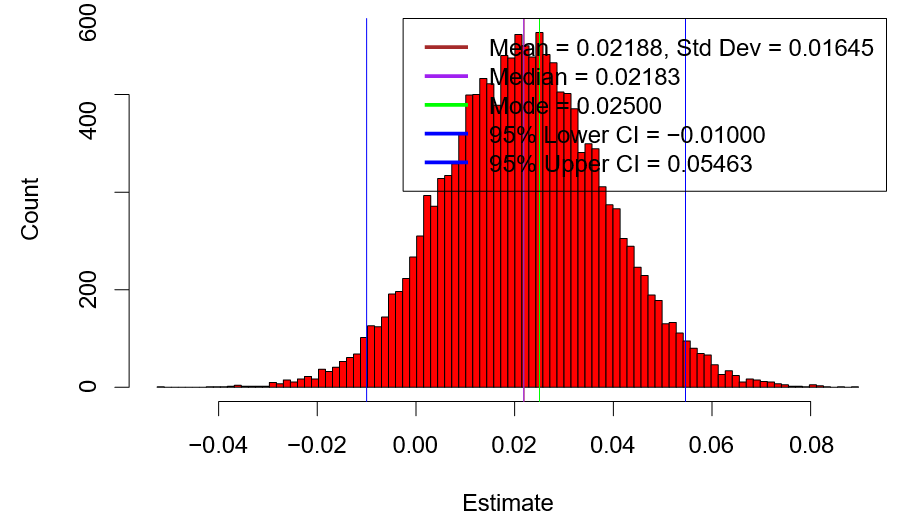


**Figure A53**

*Posterior Distribution of Level 2 Relationship: Adaptive Team Performance ON Countermeasures*


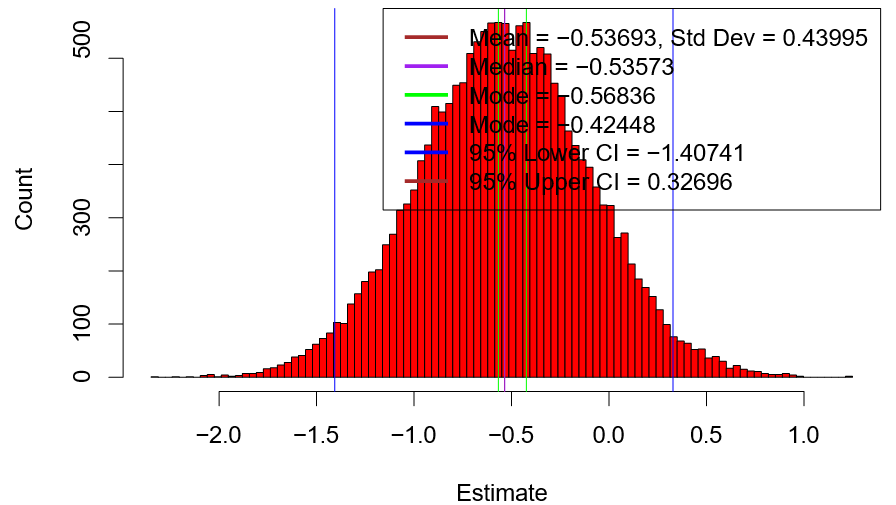


**Figure A54**

*Posterior Distribution for level-two variance for Team member fluidity*


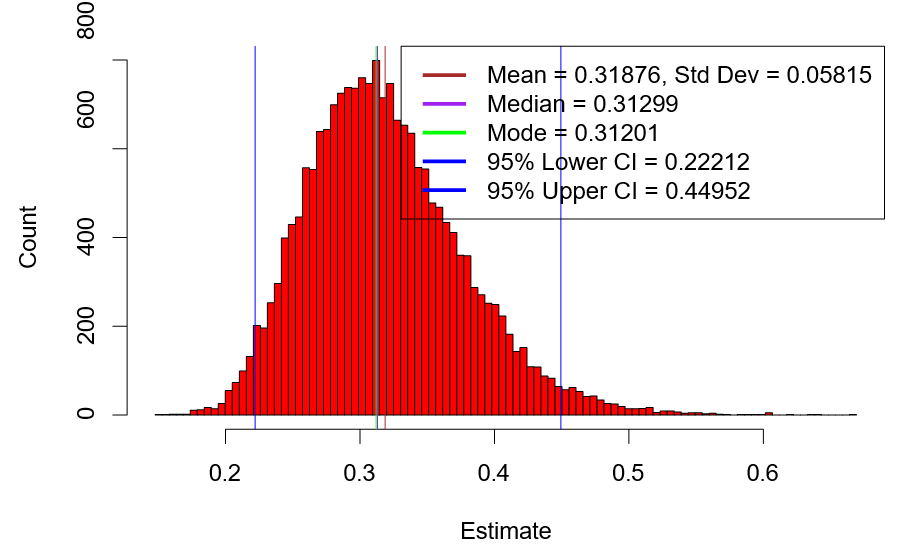


**Figure A55**

*Posterior Distribution for level-two variance for Team coordination*


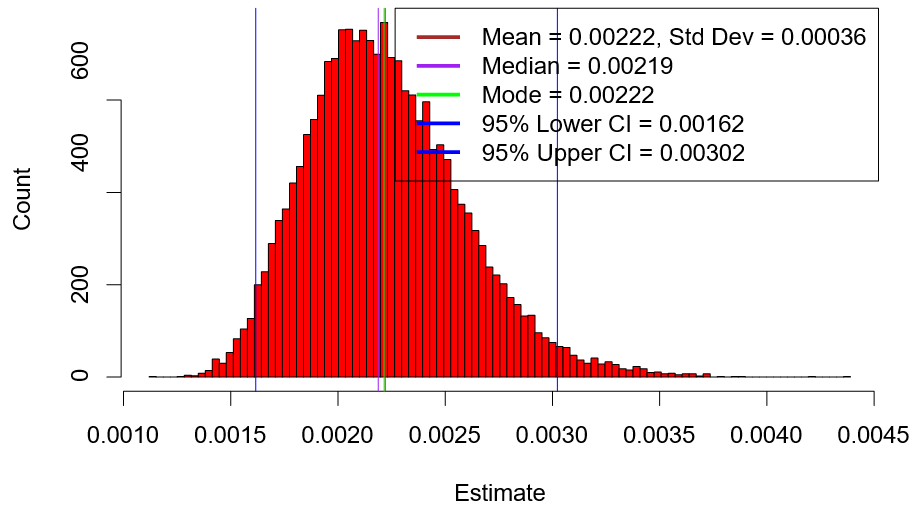


**Figure A56**

*Posterior Distribution for level-two variance of Team performance*


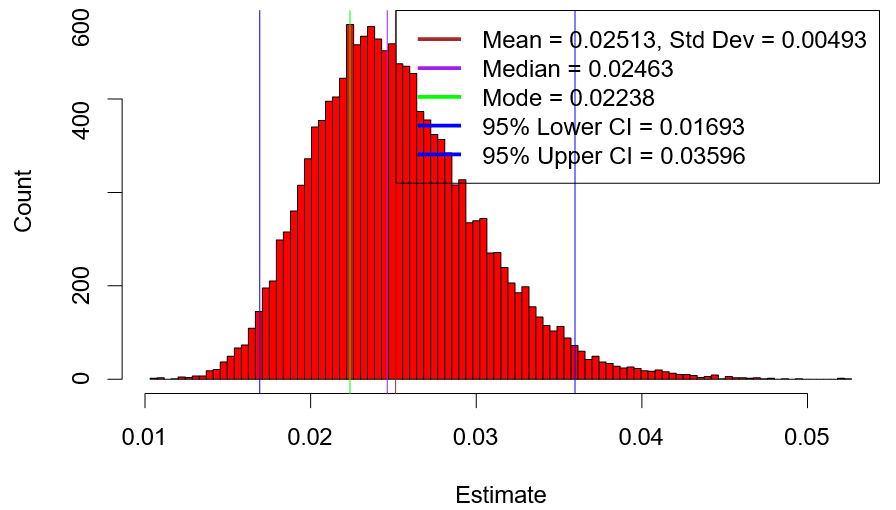


## Figure 57.

*Posterior Distribution for the team member fluidity threshold*


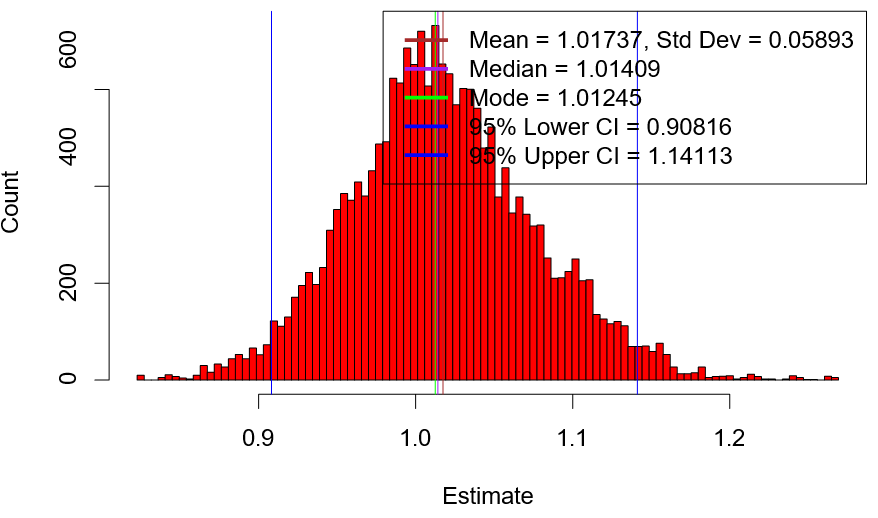


**Figure A58**

*Trace plot for the indirect effect of Adversity on team performance*


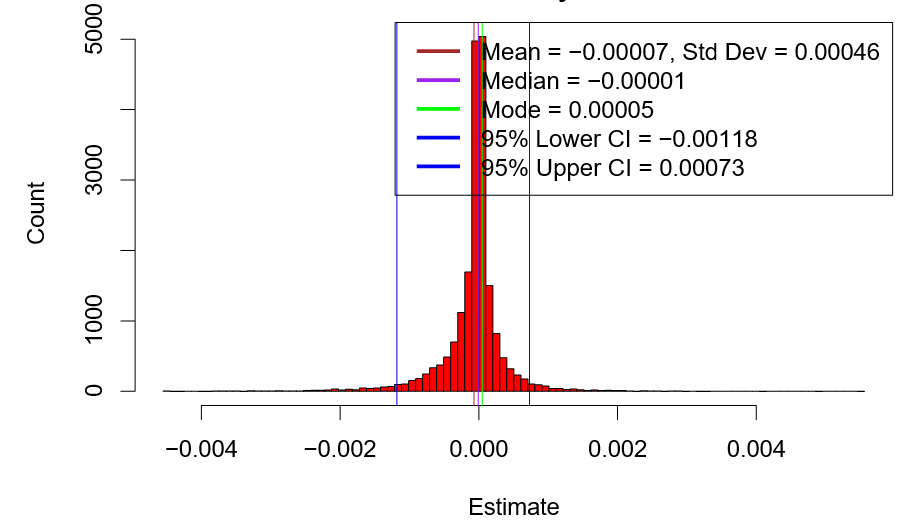


**Autocorrelation Plots with Outlier - all plots were created from the second chain**

**Figure A59**

*Autocorrelation Plot for Level-one Relationship of Team Coordination on Team Member Fluidity*


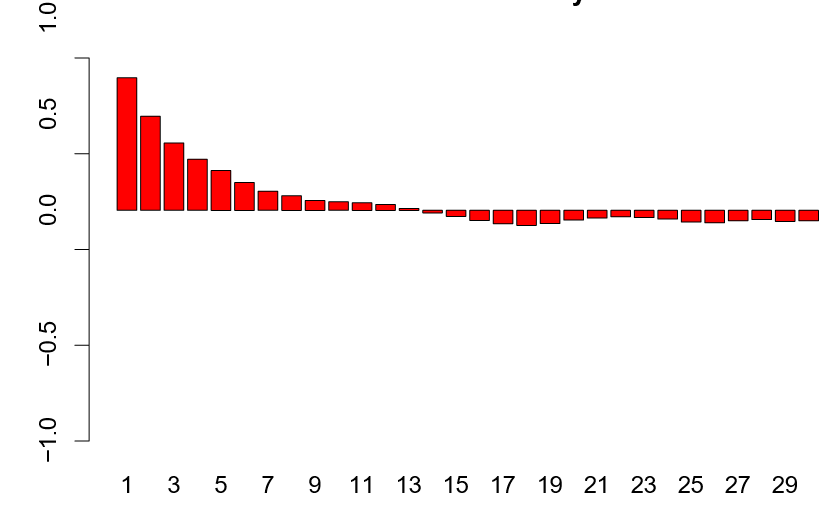


**Figure A60**

*Autocorrelation Plot for Level-one Relationship of Team Performance on Team Coordination*


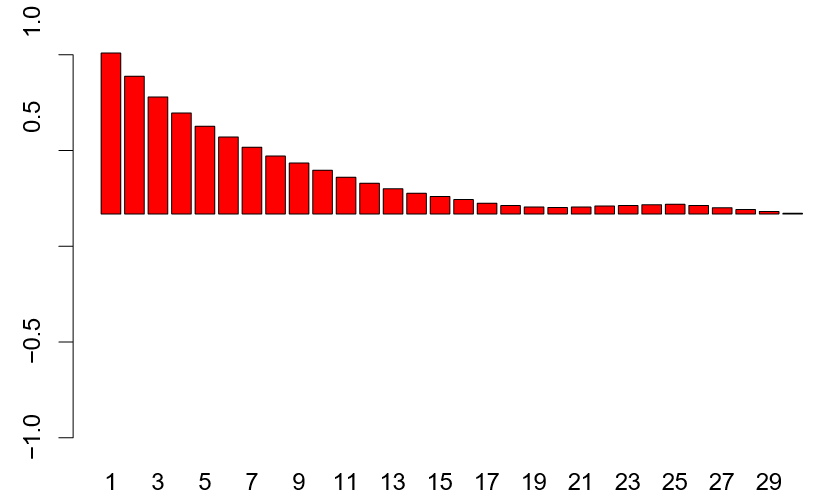


## Figure A61

*Autocorrelation plot for Level-one Variance for Team Coordination*


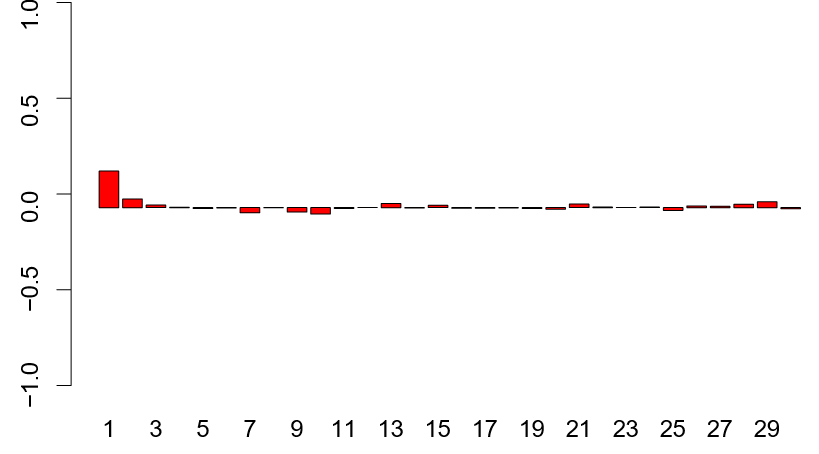


**Figure A62**

*Autocorrelation plot for level-one variance for Team Performance*


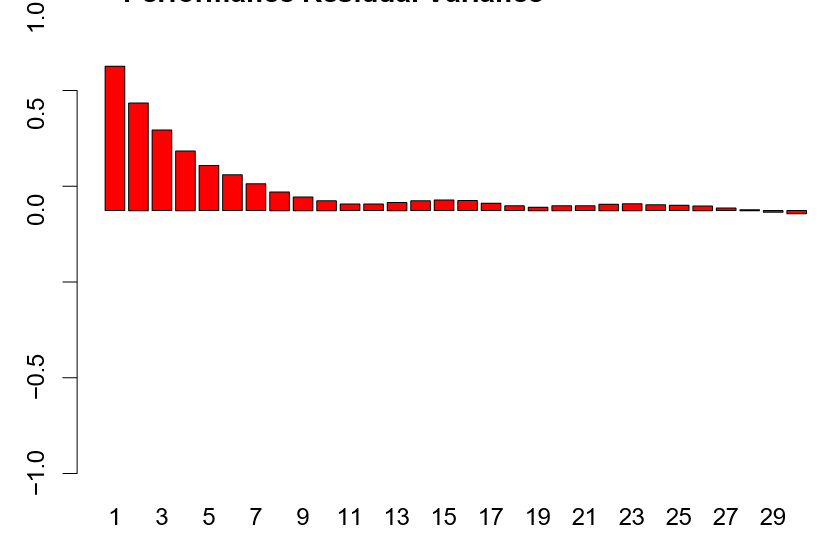


**Figure A63**

*Autocorrelation plot for the Fixed Intercept for Team Coordination*


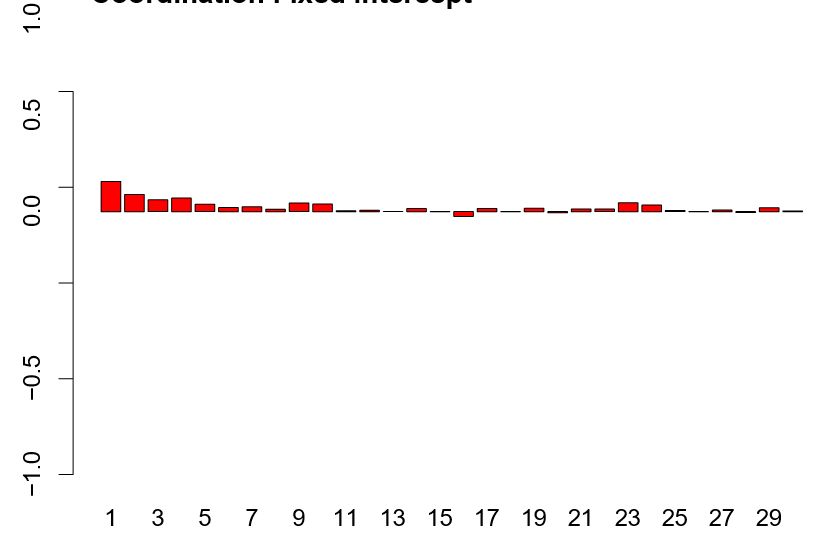


**Figure A64**

*Autocorrelation Plot for the Fixed Intercept for Team Performance*


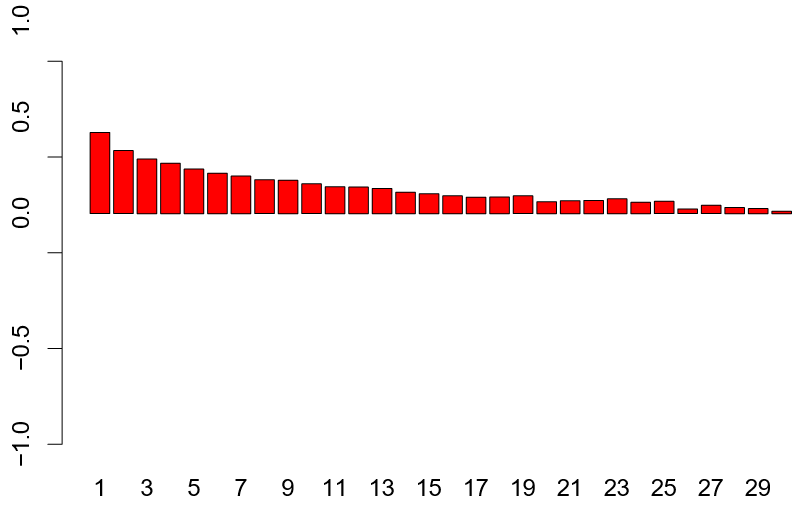


**Figure A65**

*Autocorrelation Plot for the Level 2 Relationship: Team member fluidity ON Adversity*


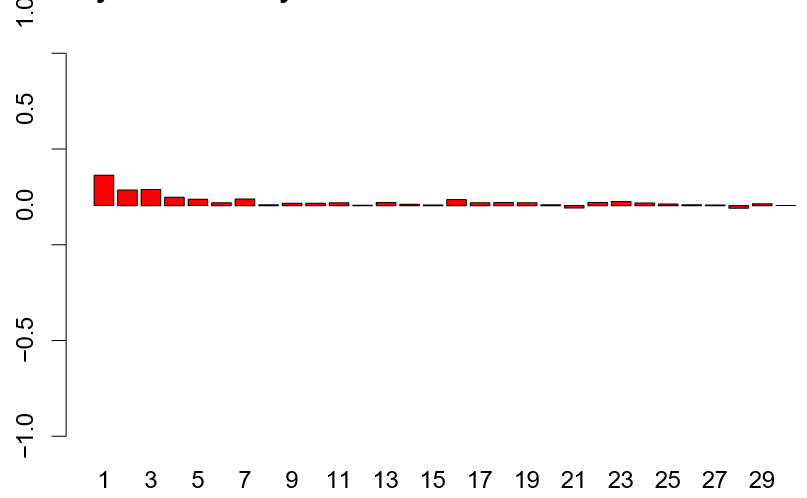


**Figure A66**

*Autocorrelation Plot of Level 2 Relationship: Team member fluidity ON Countermeasures*


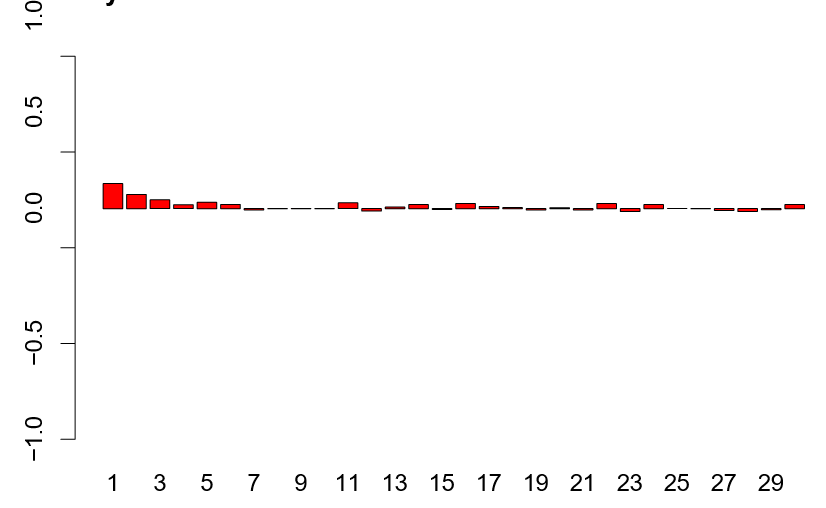


**Figure A67**

*Autocorrelation Plot of Level 2 Relationship: Team coordination ON Team member fluidity*


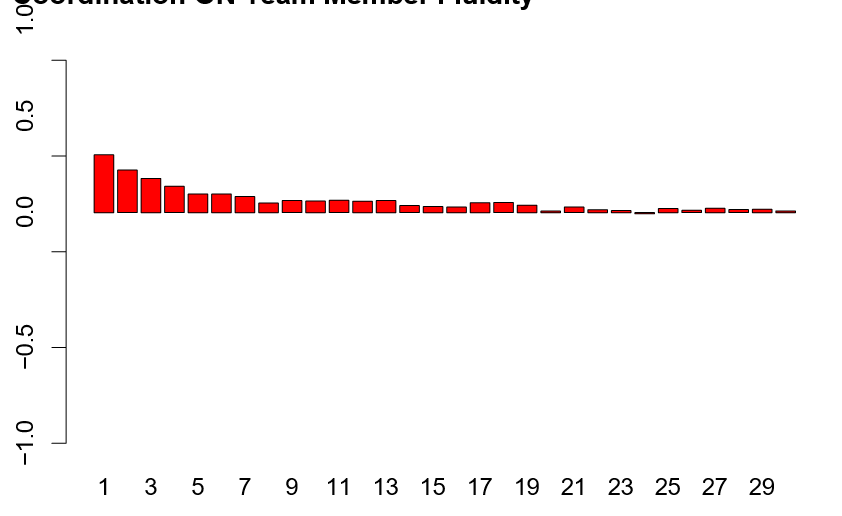


**Figure A68**

*Autocorrelation Plot of Level 2 Relationship: Adaptive Team Performance ON Team Coordination*


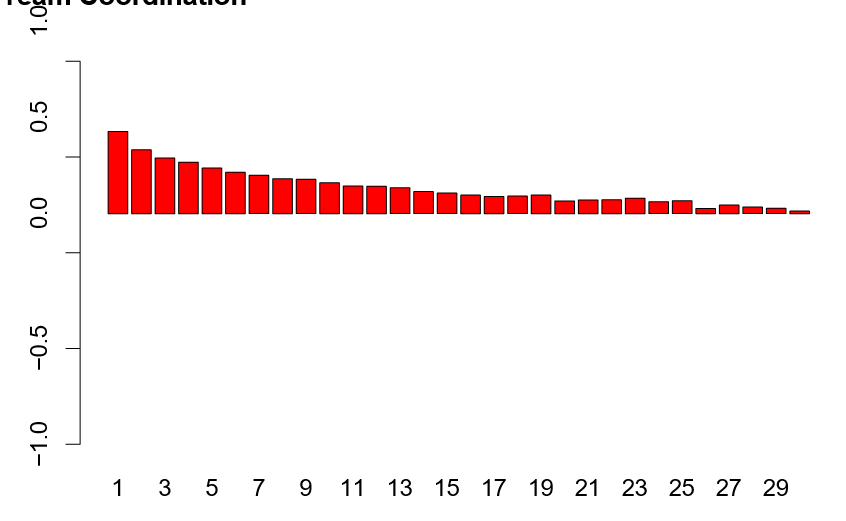


**Figure A69**

*Autocorrelation Plot for the Level 2 Relationship: Team Performance ON Adversity*


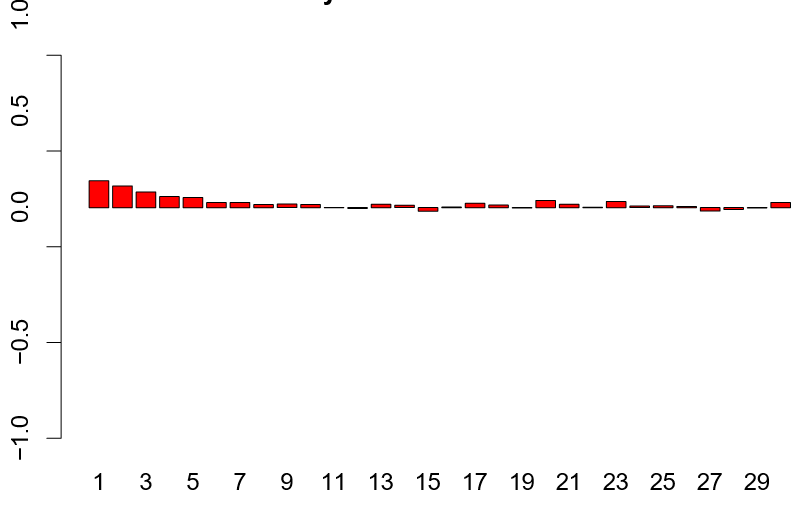


**Figure A70**

*Autocorrelation plot for the Level 2 Relationship: Team Performance ON Countermeasures*


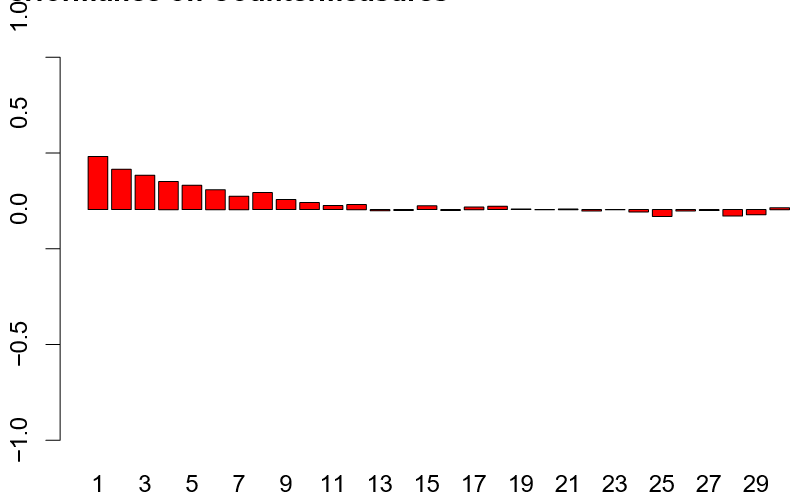


**Figure A71**

*Autocorrelation Plot for the level-two variance for Team member fluidity*


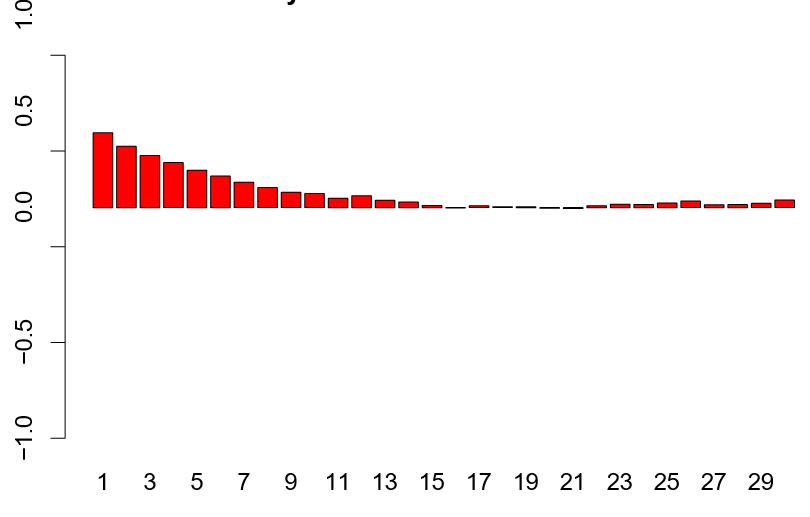


**Figure A72**

*Autocorrelation Plot for the level-two variance for Team coordination*


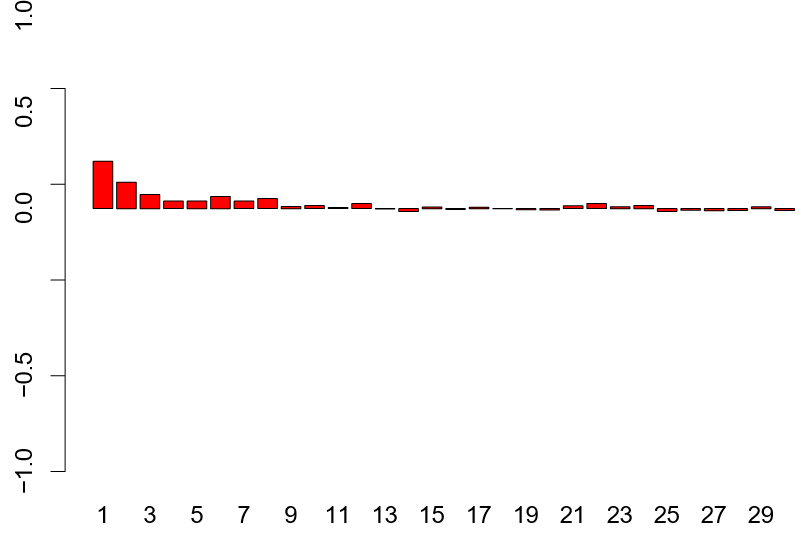


**Figure A73**

*Autocorrelation Plot for the level-two variance for team performance*


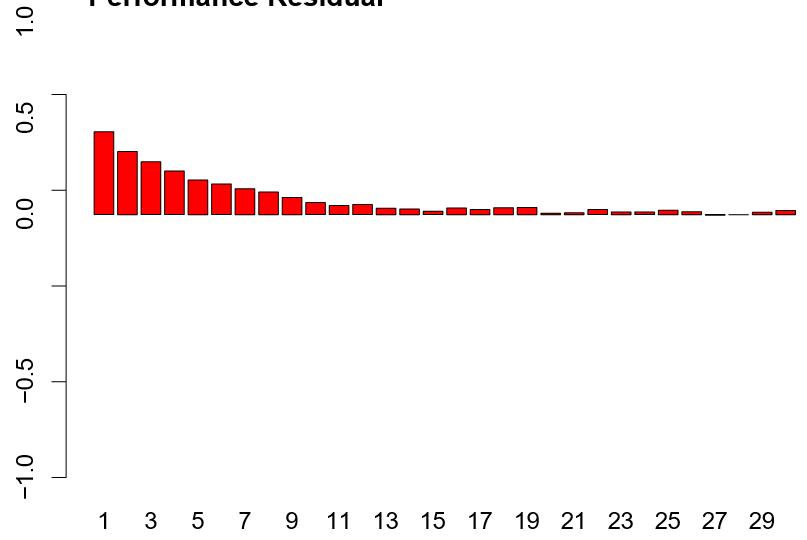


**Figure A74**

*Autocorrelation plot for the team member fluidity threshold*


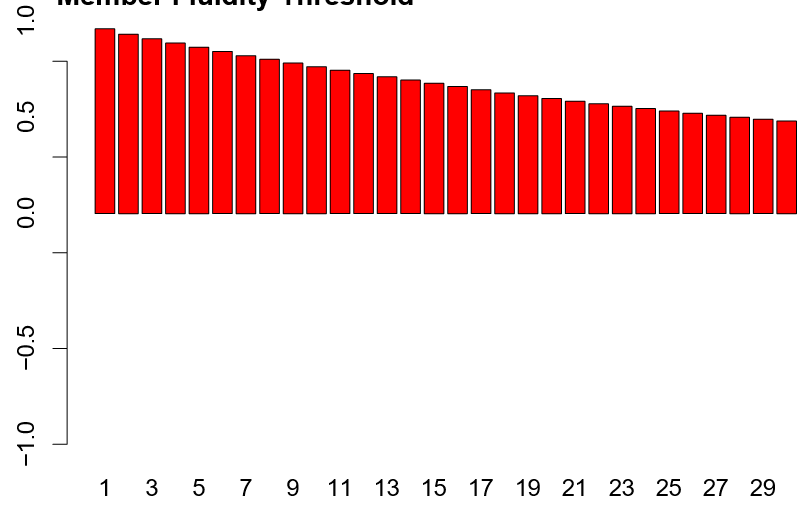


**Figure A75**

*Autocorrelation plot for the indirect effect of Adversity on team performance*

*
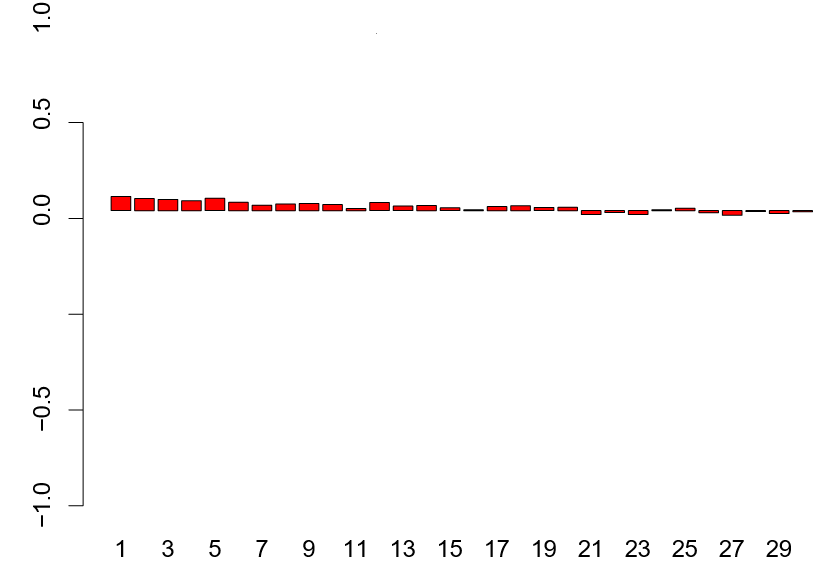
*

# Hypotheses 2 and 3 without Outlier

## Trace Plots without Outliers

**Figure A76**

*Trace Plot for Level-one Relationship of Team Coordination on Team Member Fluidity*


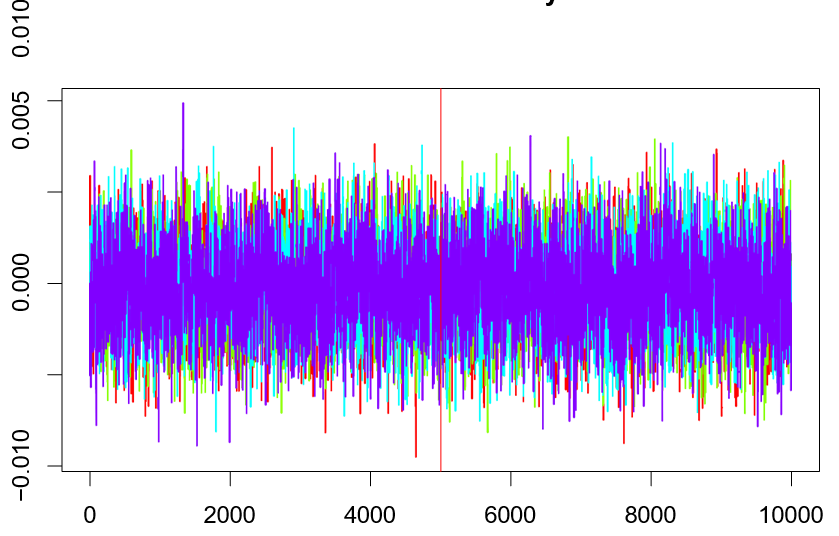


**Figure A77**

*Trace Plot for Level-one Relationship of Team Performance on Team Coordination*


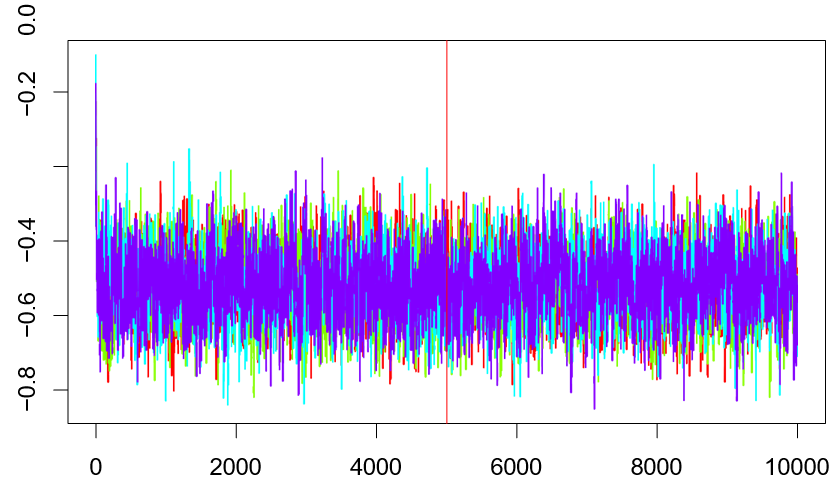


**Figure A78**

*Trace Plot for Level-one Variance for Team Coordination*


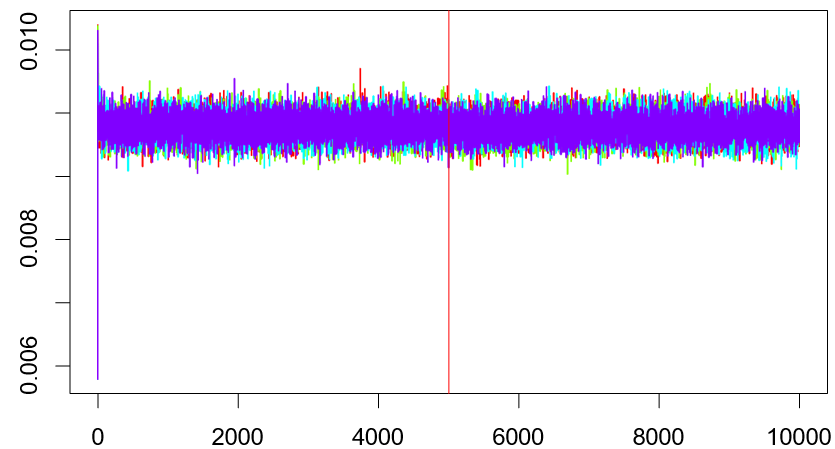


**Figure A79**

*Trace plot for level-one variance for Team Performance*


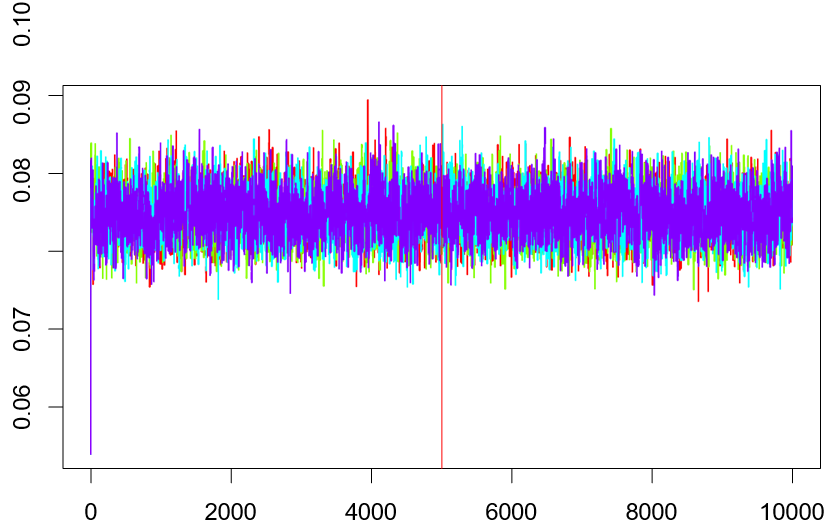


**Figure A80**

*Trace Plot for the Fixed Intercept for Team Coordination*


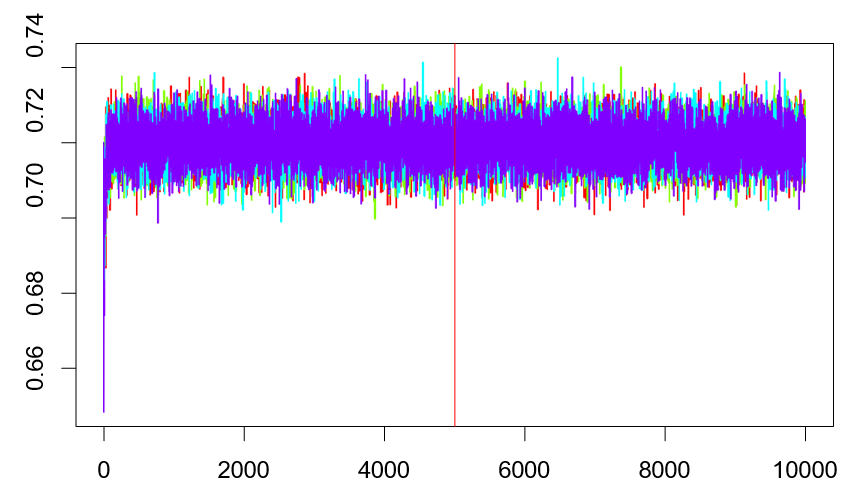


**Figure A81**

*Trace plot for the fixed intercept for Team Performance*


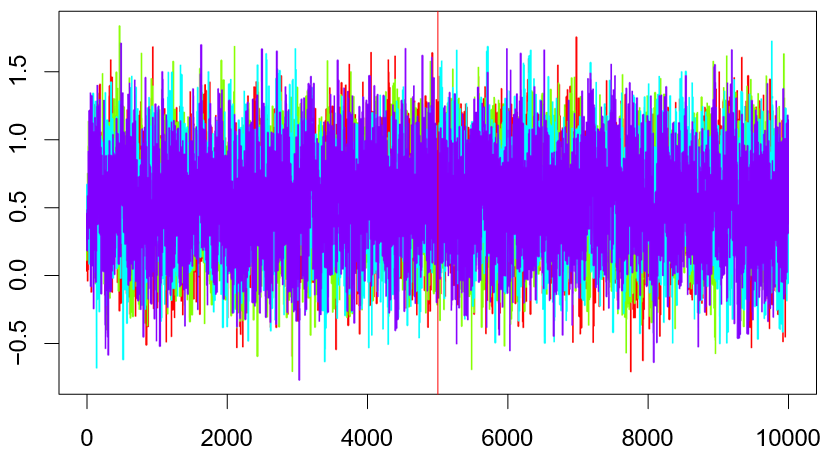


**Figure A82**

*Trace plot of Level 2 Relationship: Team member fluidity ON Adversity*


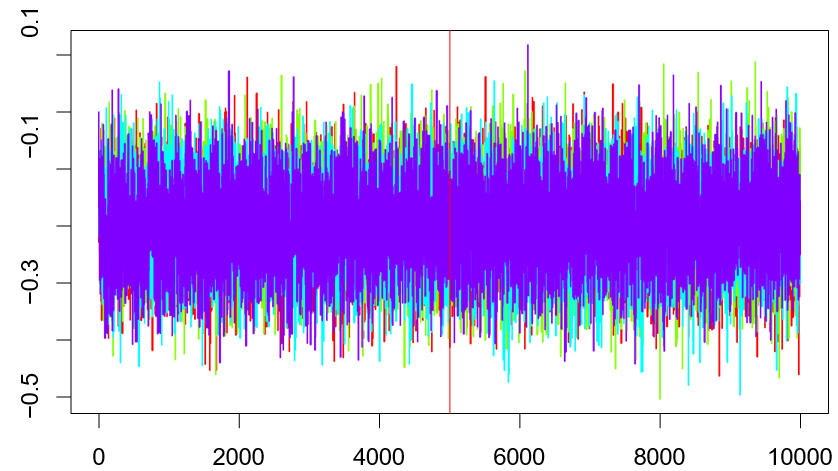


**Figure A83**

*Trace plot of Level 2 Relationship: Team member fluidity ON Countermeasures*


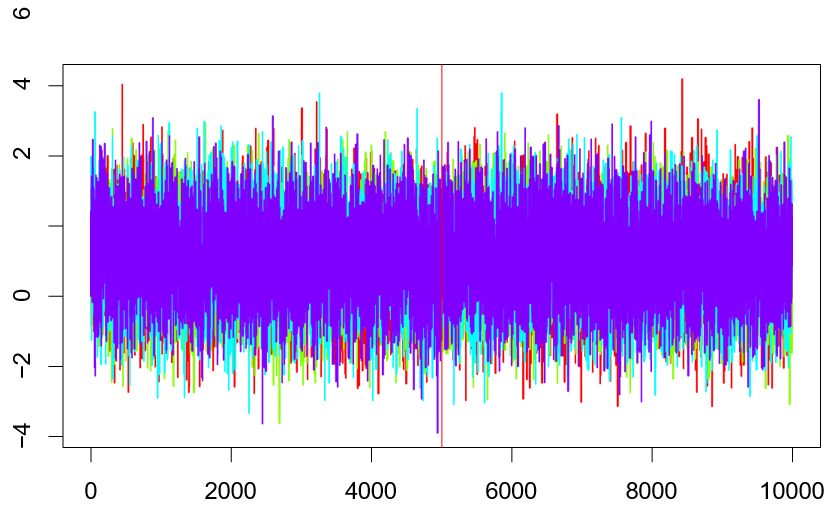


**Figure A84**

*Trace plot of Level 2 Relationship: Team coordination ON team member fluidity*


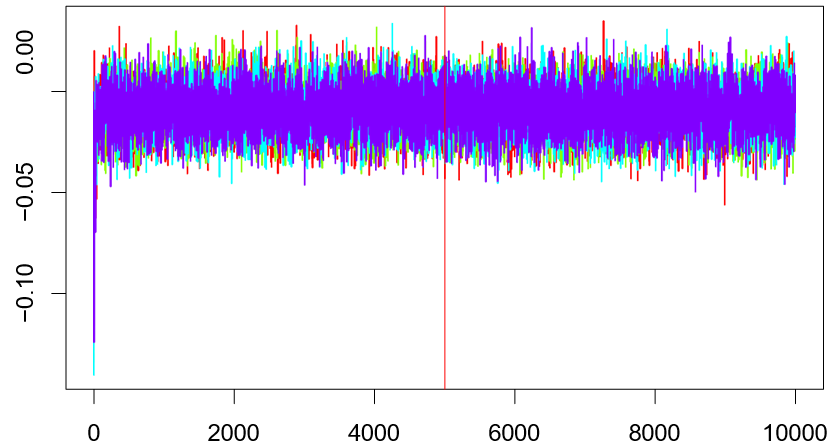


**Figure A85**

*Trace plot of Level 2 Relationship: Team Performance ON Team Coordination*


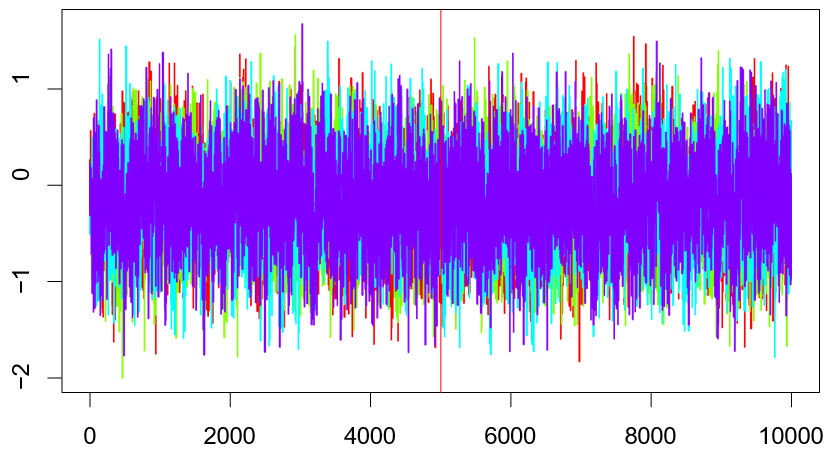


**Figure A86**

*Trace plot of Level 2 Relationship: Adaptive Team Performance ON Adverstiy*


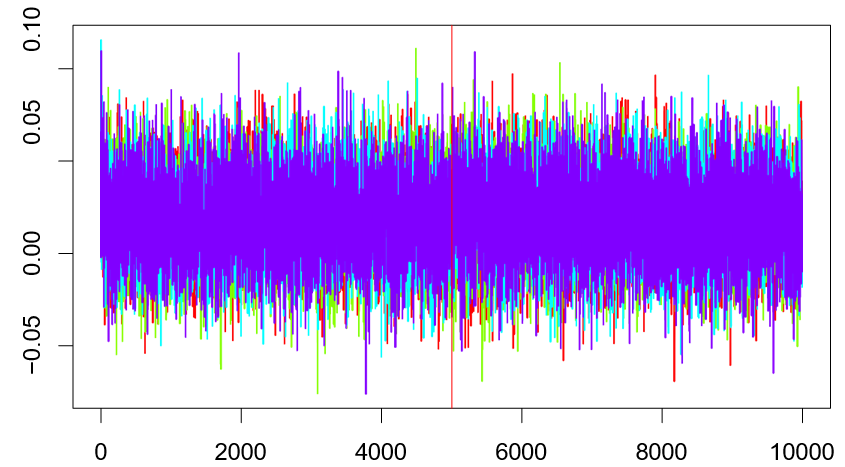


**Figure A87**

*Trace plot of Level 2 Relationship: Team Performance ON Countermeasures*


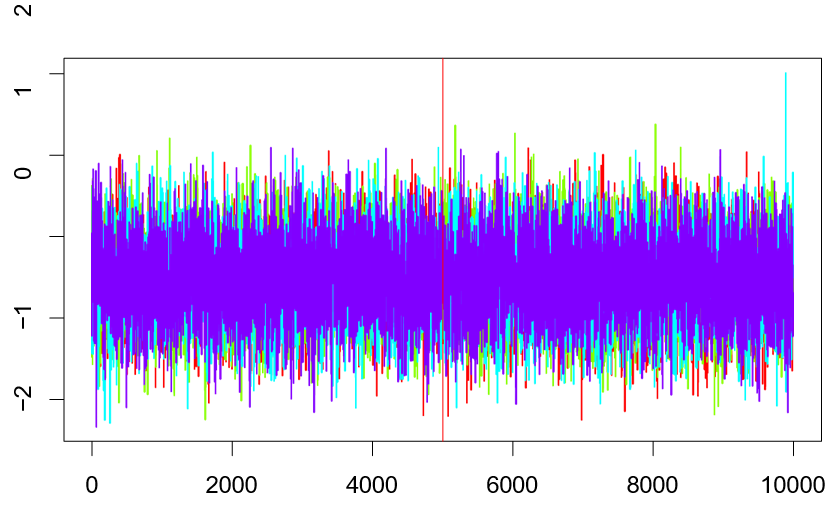


**Figure A88**

*Trace plot for level-two variance for Team member fluidity*


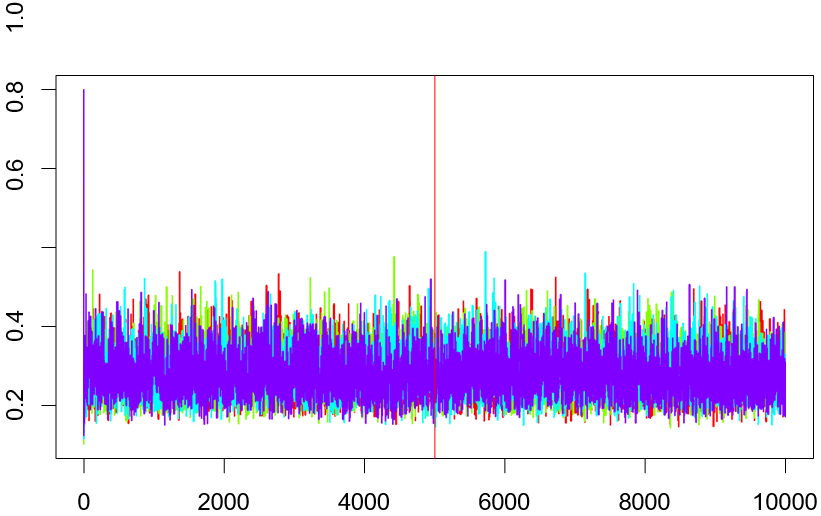


**Figure A89**

*Trace plot for level-two variance for Team coordination*


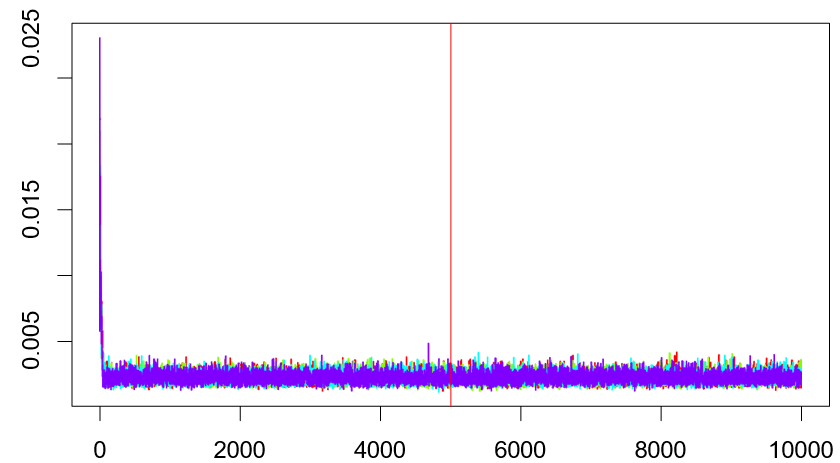


**Figure A90**

*Trace plot for level-two variance for adaptive team performance*


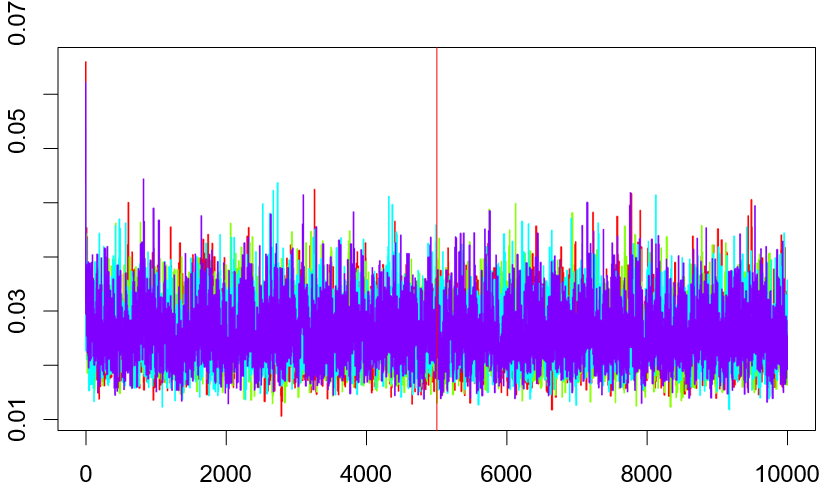


**Figure A91**

*Trace plot for the team member fluidity threshold*


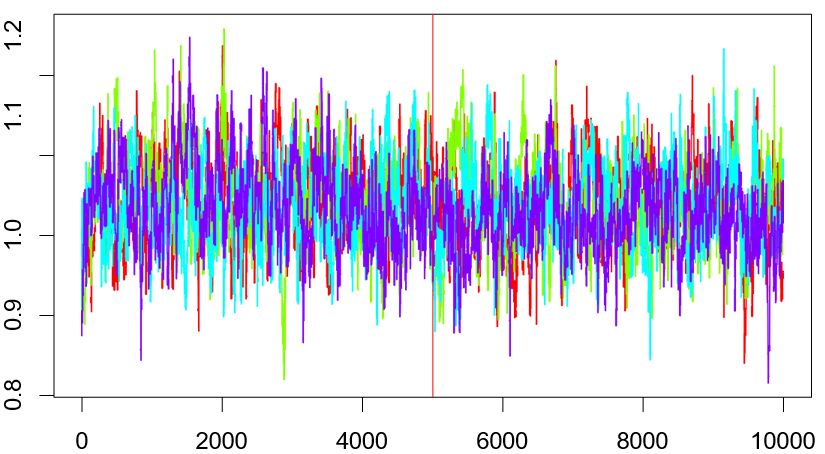


**Figure A92**

*Trace plot for the indirect effect of Adversity on team performance*

**
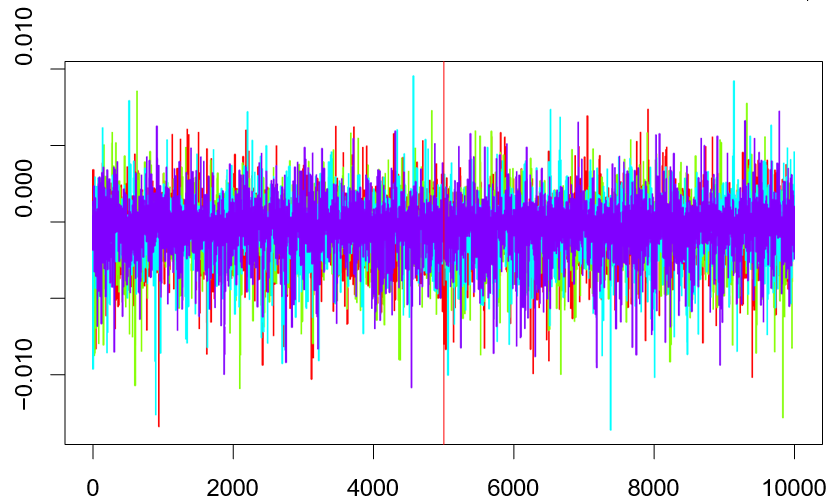
**

**Posterior Distributions for the Parameters without Outliers**

**Figure A93**

*Posterior Distribution for Level-one Relationship of Team Coordination on Team Member Fluidity*


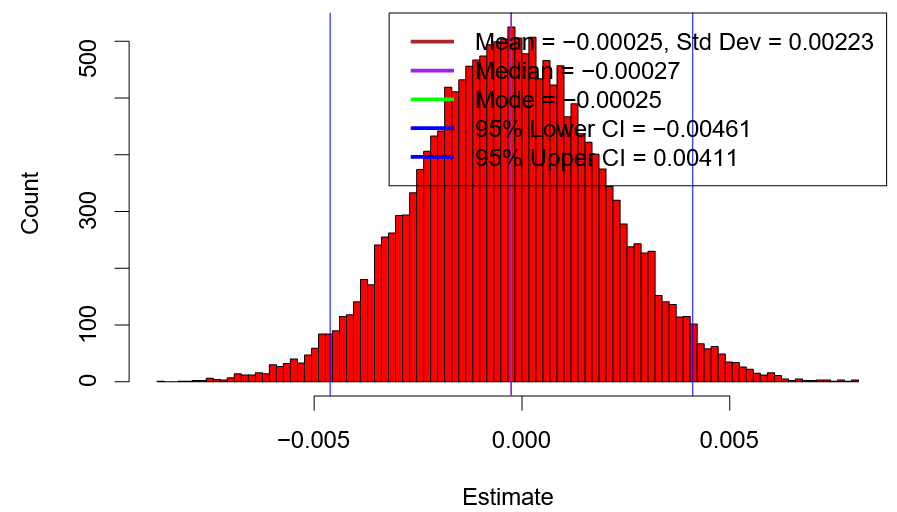


**Figure A94**

*Posterior Distribution for Level-one Relationship of Adaptive Team Performance on Team Coordination*


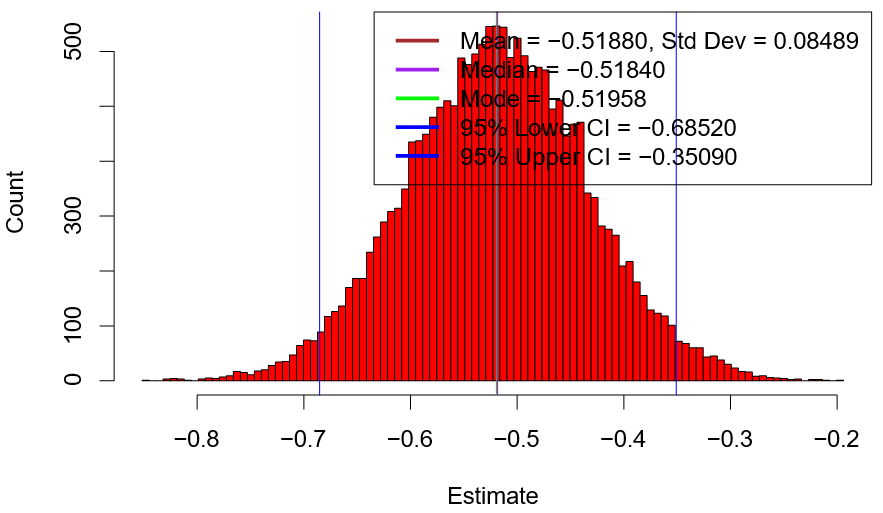


**Figure A95**

*Posterior Distribution for Level-one Variance for Team Coordination*


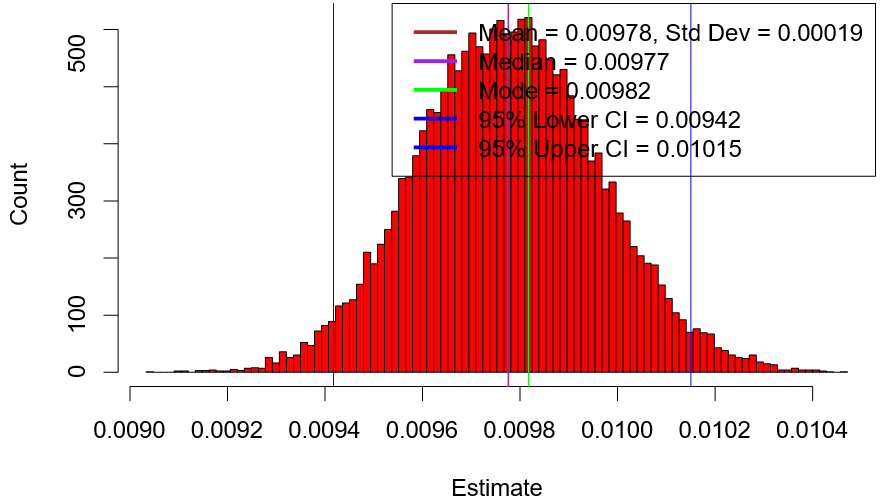


**Figure A96**

*Posterior Distribution for level-one variance for Team Performance*


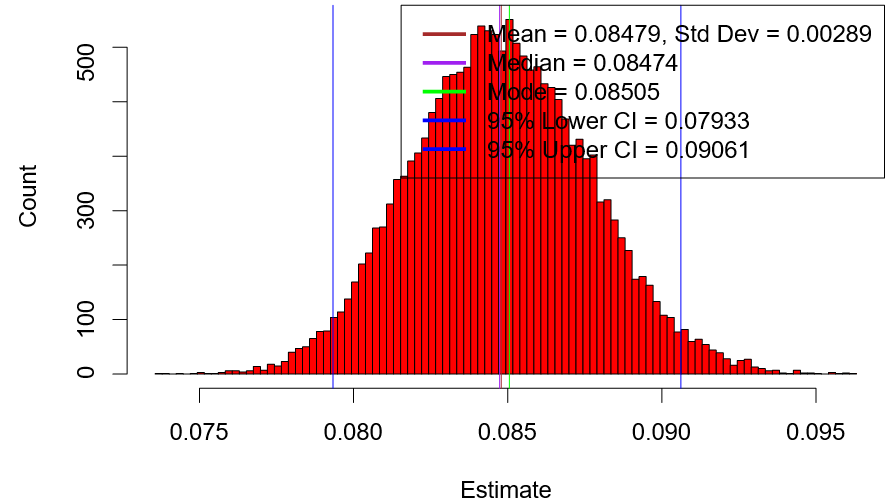


**Figure A97**

*Posterior Distribution for the Fixed Intercept for Team Coordination*


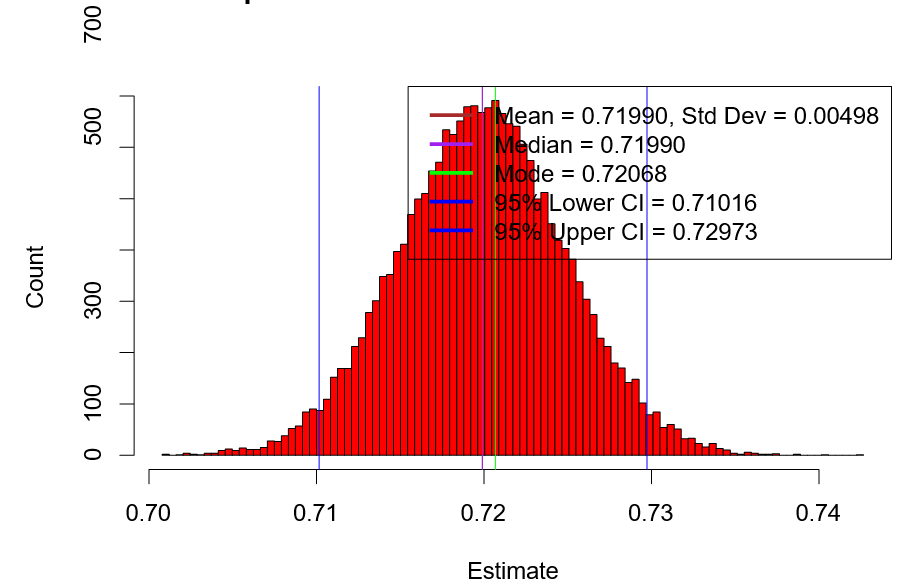


**Figure A98**

*Posterior Distribution for the fixed intercept for Team Performance*


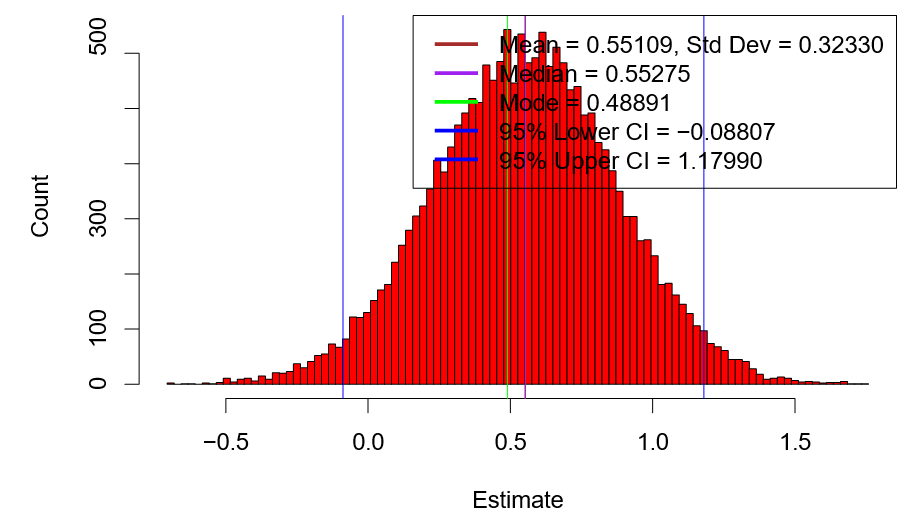


**Figure A99**

*Posterior Distribution of Level 2 Relationship: Team member fluidity ON Adversity*


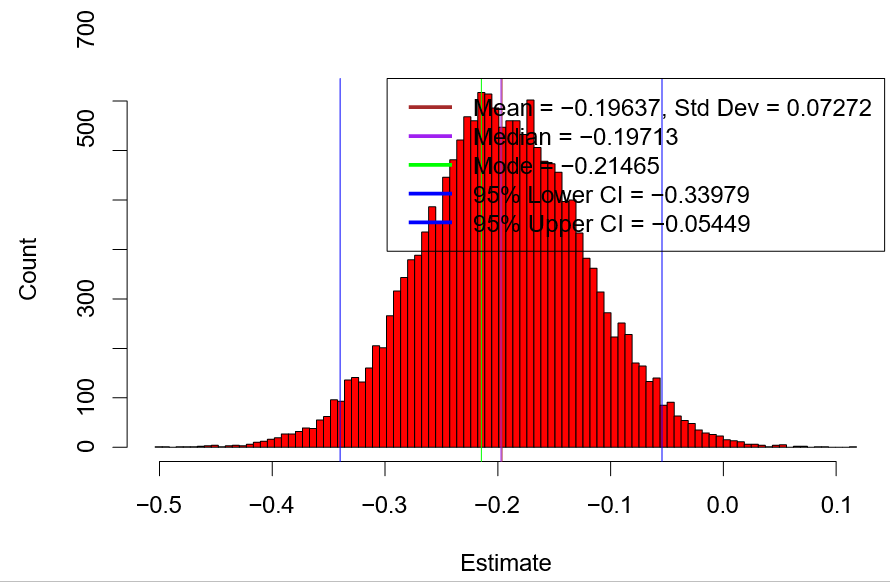


**Figure A100**

*Posterior Distribution of Level 2 Relationship: Team member fluidity ON Countermeasures*


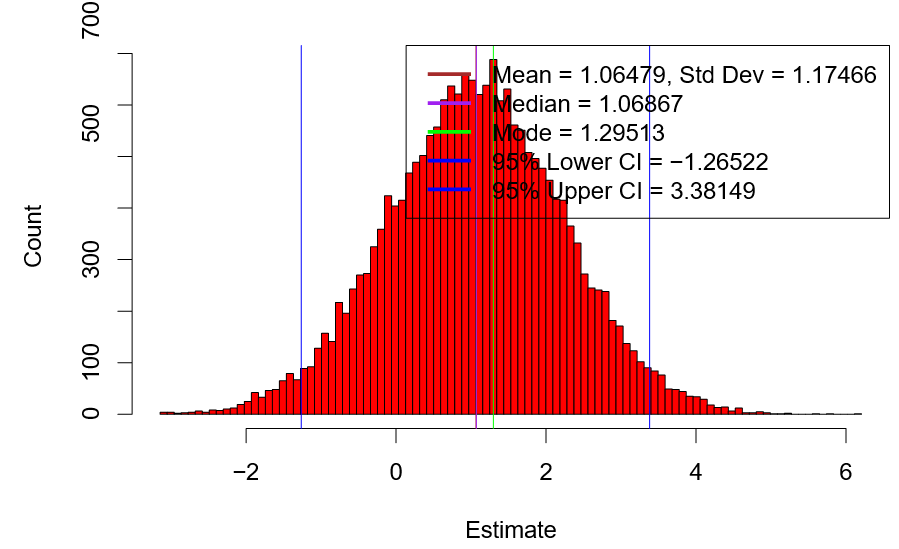


**Figure A101**

*Posterior Distribution of Level 2 Relationship: Team coordination ON team member fluidity*

**Figure A102**

*Posterior Distribution of Level 2 Relationship: Adaptive Team Performance ON Team Coordination*

**Figure A103**

*Posterior Distribution of Level 2 Relationship: Team Performance ON Adversity*

**Figure A104**

*Posterior Distribution of Level 2 Relationship: Team Performance ON Countermeasures*

**Figure A105**

*Posterior Distribution for level-two variance for Team member fluidity*

**Figure A106**

*Posterior Distribution for level-two variance for Team Coordination*

**Figure A107**

*Posterior Distribution for level-two variance for team performance*

**Figure A108**

*Posterior Distribution for the team member fluidity threshold*

**Figure A109**

*Posterior Distribution for the indirect effect of Adversity on team performance*

**Autocorrelation Plots without Outlier**

**Figure A110**

*Autocorrelation Plot for Level-one Relationship of Team Coordination on Team Member Fluidity*

**Figure A111**

*Autocorrelation Plot for Level-one Relationship of Team Performance on Team Coordination*

**Figure** A112

*Autocorrelation plot for Level-one Variance for Team Coordination*

**Figure A113**

*Autocorrelation plot for level-one variance for Team Performance*

**Figure A114**

*Autocorrelation Plot for the Fixed Intercept for Team Coordination*

**Figure A115**

*Autocorrelation Plot for the fixed intercept for Team Performance*

**Figure A116**

*Autocorrelation Plot for the Level 2 Relationship: Team member fluidity ON Adversity*

**Figure A117**

*Autocorrelation Plot of Level 2 Relationship: Team member fluidity ON Countermeasures*

**Figure A118**

*Autocorrelation Plot of Level 2 Relationship: Team coordination ON team member fluidity*

**Figure A119**

*Autocorrelation Plot of Level 2 Relationship: Team Performance ON Team Coordination*

**Figure A120**

*Autocorrelation Plot for the Level 2 Relationship: Team Performance ON Adversity*

**Figure A121**

*Autocorrelation plot for the Level 2 Relationship: Team Performance ON Countermeasures*

**Figure A122**

*Autocorrelation Plot for the level-two variance for Team member fluidity*

**Figure A123**

*Autocorrelation Plot for the level-two variance for Team coordination*

**Figure A124**

*Autocorrelation Plot for the level-two variance for team performance*

**Figure A125**

*Autocorrelation plot for the team member fluidity threshold*

**Figure A126**

*Autocorrelation plot for the indirect effect of Adversity on team performance*

**Hypothesis 4 with Outlier**

**Trace Plots with Outlier**

**Figure A127**

*Trace Plot for Level-one Relationship of Team Coordination on Team Member Fluidity*

**Figure A128**

*Trace Plot for Level-one Relationship of Team Performance on Team Coordination*

**Figure A129**

*Trace Plot for Level-one Variance for Team Coordination*

**Figure A130**

*Trace plot for level-one variance for Team Performance*

**Figure A131**

*Trace Plot for the Fixed Intercept for Team Coordination*

**Figure A132**

*Trace plot for the fixed intercept for Team Performance*

**Figure A133**

*Trace plot of Level 2 Relationship: Team member fluidity ON Adversity*

**Figure A134**

*Trace plot of Level 2 Relationship: Team member fluidity ON Countermeasures*

**Figure A135**

*Trace plot of Level 2 Relationship: Team member fluidity ON Adversity*

**Figure A136**

*Trace plot of Level 2 Relationship: Team coordination ON team member fluidity*

**Figure A137**

*Trace plot of Level 2 Relationship: Team Performance ON Team Coordination*

**Figure A138**

*Trace plot of Level 2 Relationship: Adaptive Team Performance ON Adversity*

**Figure A139**

*Trace plot of Level 2 Relationship: Team Performance ON Countermeasures*

**Figure A140**

*Trace plot of Level 2 Relationship: Team Performance ON Adversity by Countermeasures Interaction*

**Figure A141**

*Trace plot for level-two variance for Team member fluidity*

**Figure A142**

*Trace plot for level-two variance for Team coordination*

**Figure A143**

*Trace plot for level-two variance for Team performance*

**Figure A171**

*Trace plot for the team member fluidity threshold*

**Figure A172**

*Trace plot for the indirect effect of Adversity on team performance*

**Posterior Distributions for the Parameters with Outlier**

**Figure A146**

*Posterior Distribution for Level-one Relationship of Team Coordination on Team Member Fluidity*

**Figure A147**

*Posterior Distribution for Level-one Relationship of Adaptive Team Performance on Team Coordination*

**Figure A148**

*Posterior Distribution for Level-one Variance for Team Coordination*

**Figure A149**

*Posterior Distribution for level-one variance for Team Performance*

**Figure A150**

*Posterior Distribution for the Fixed Intercept for Team Coordination*

**Figure A151**

*Posterior Distribution for the fixed intercept for Team Performance*

**Figure A152**

*Posterior Distribution of Level 2 Relationship: Team member fluidity ON Adversity*

**Figure A153**

*Posterior Distribution of Level 2 Relationship: Team member fluidity ON Countermeasures*

**Figure A154**

*Posterior Distribution of Level 2 Relationship: Team member fluidity ON Adversity by Countermeasures Interaction*

**Figure A155**

*Posterior Distribution of Level 2 Relationship: Team coordination ON team member fluidity*

**Figure A156**

*Posterior Distribution of Level 2 Relationship: Team Performance ON Team Coordination*

**Figure A157**

*Posterior Distribution of Level 2 Relationship: Adaptive Team Performance ON Adversity*

**Figure A158**

*Posterior Distribution of Level 2 Relationship: Adaptive Team Performance ON Countermeasures*

**Figure A159**

*Posterior Distribution of Level 2 Relationship: Team Performance ON Adversity by Countermeasures Interaction*

**Figure A160**

*Posterior Distribution for level-two variance for Team member fluidity*

**Figure A161**

*Posterior Distribution for level-two variance for Team coordination*

**Figure A162**

*Posterior Distribution for level-two variance for team performance*

**Figure A163**

*Posterior Distribution for the team member fluidity threshold*

**Figure A164**

*Posterior Distribution for the indirect effect of Adversity on team performance*

**Autocorrelation Plots with Outlier**

**Figure A165**

*Autocorrelation Plot for Level-one Relationship of Team Coordination on Team Member Fluidity*

**Figure A166**

*Autocorrelation Plot for Level-one Relationship of Adaptive Team Performance on Team Coordination*

**Figure A167**

*Autocorrelation plot for Level-one Variance for Team Coordination*

**Figure A168**

*Autocorrelation plot for level-one variance for Team Performance*

**Figure A169**

*Autocorrelation Plot for the Fixed Intercept for Team Coordination*

**Figure A170**

*Autocorrelation Plot for the fixed intercept for Team Performance*

**Figure A171**

*Autocorrelation Plot for the Level 2 Relationship: Team member fluidity ON Adversity*

**Figure A172**

*Autocorrelation Plot of Level 2 Relationship: Team member fluidity ON Countermeasures*

**Figure A173**

**Figure A174**

**Figure A175**

**Figure A176**

**Figure A177**

**Figure A178**

1 2 3 4 5 6 7 8 9 10 11 12 13 14 15 16 17 18 19 20 21 22 23 24 25 26 27 28 29 30

−1.0

−0.5

**Figure A179**

**Figure A180**

**Figure A181**

**Figure A182**

**Figure A183**
